# Rapid generation of purified human RPE from pluripotent stem cells using 2D cultures and lipoprotein uptake-based sorting

**DOI:** 10.1186/s13287-020-1568-3

**Published:** 2020-02-03

**Authors:** Fabio Michelet, Aishwarya Balasankar, Nickolas Teo, Lawrence W. Stanton, Shweta Singhal

**Affiliations:** 1 Singapore Eye Research Institute, Singapore National Eye Centre, Singapore, Singapore; 2 Duke-NUS, Medical School, Singapore, Singapore; 3 Stem Cell and Regenerative Biology Group, Genome Institute of Singapore, Singapore, Singapore; 4 Qatar Biomedical Research Institute, Hamad Bin Khalifa University, Doha, Qatar; 5 Yong Loo Lin School of Medicine, National University of Singapore, Singapore, Singapore

## Abstract

**Background:**

Despite increasing demand, current protocols for human pluripotent stem cell (hPSC)-derived retinal pigment epithelium (RPE) remain time, labor, and cost intensive. Additionally, absence of robust methods for selective RPE purification and removal of non-RPE cell impurities prevents upscaling of clinical quality RPE production. We aimed to address these challenges by developing a simplified hPSC-derived RPE production and purification system that yields high-quality RPE monolayers within 90 days.

**Methods:**

Human pluripotent stem cells were differentiated into RPE using an innovative time and cost-effective protocol relying entirely on 2D cultures and minimal use of cytokines. Once RPE identity was obtained, cells were transferred onto permeable membranes to acquire mature RPE morphology. RPE differentiation was verified by electron microscopy, polarized VEGF expression, establishment of high transepithelial electrical resistance and photoreceptor phagocytosis assay. After 4 weeks on permeable membranes, RPE cell cultures were incubated with Dil-AcLDL (DiI-conjugated acetylated low-density lipoproteins) and subjected to fluorescence-activated cell sorting (FACS) for purification and subculture.

**Results:**

Using our 2D cytokine scarce protocol, hPSC-derived functional RPE cells can be obtained within 2 months. Nevertheless, at this stage, most samples contain a percentage of non-RPE/early RPE progenitor cells that make them unsuitable for clinical application. We demonstrate that functional RPE cells express high levels of lipoprotein receptors and that this correlates with their ability to uptake lipoproteins. Combining photoreceptor uptake assay with lipoprotein uptake assay further confirms that only functional RPE cells uptake AcLDL. Incubation of mixed RPE/non-RPE cell cultures with fluorophore conjugated AcLDL and subsequent FACS-based isolation of labeled cells allows selective purification of mature functional RPE. When subcultured, DiI-AcLDL-labeled cells rapidly form pure homogenous high-quality RPE monolayers.

**Conclusions:**

Pure functional RPE monolayers can be derived from hPSC within 90 days using simplified 2D cultures in conjunction with our RPE PLUS protocol (RPE Purification by Lipoprotein Uptake-based Sorting). The simplicity of this protocol makes it scalable, and the rapidity of production and purification allows for high-quality RPE to be produced in a short span of time making them ideally suited for downstream clinical and in vitro applications.

**Electronic supplementary material:**

The online version of this article (10.1186/s13287-020-1568-3) contains supplementary material, which is available to authorized users.

## Background

Age-related macular degeneration (AMD) is responsible for 7–8% of blindness worldwide and is the most common cause of blindness in developed countries, with projected number of patients reaching 200 million by 2020 [1, 2]. Retinal pigment epithelium (RPE) dysfunction is the key pathological process in AMD and blindness is caused by photoreceptor loss that occurs secondary to RPE dysfunction [3]. Over the last decade, significant progress has been made in the field of cell replacement therapy for AMD using stem cell-derived RPE [4]. Pluripotent stem cells such as human embryonic stem cells (hESCs) or human-induced pluripotent stem cells (hiPSCs) can now be consistently differentiated into functional RPE in vitro and their use in patients with AMD in clinical trials shows promising functional recovery [5–9]. In addition, stem cell-derived RPE are also valuable as models to study pathogenesis of retinal diseases and assess treatment response in vitro [10–12]. This has led to an unprecedented demand, both clinically and in research, for production of high-quality stem cell-derived RPE.

Most current protocols to generate RPE from pluripotent stem cells use a two-step strategy that includes induction (pluripotent stem cells induced to form retinal progenitors) followed by expansion and differentiation (retinal progenitors pushed to form functional RPE) [13, 14]. During induction, small clusters of stem cell colonies are cultured in suspension or embedded in extracellular matrix protein gels with a cocktail of cytokines [10, 15, 16]. In these conditions, the pluripotent stem cells first begin to form embryoid bodies or neuroepithelial cysts. The retinal progenitors contained in these structures subsequently begin to form pigmented RPE-like cells over a period of 4 to 22 weeks depending on the protocol in question. Once pigmentation has been induced, the next step involves manual selection and isolation of pigmented patches, their dissociation into single-cell suspensions, replating for further expansion, and RPE differentiation. Some protocols use 2D strategies and directed differentiation by specific cocktails of cytokines to be added in a precise sequence during the differentiation process. These protocols are expensive and result in RPE monolayers that contain significant numbers of non-RPE cells [12, 17, 18]. When the resulting RPE monolayer is not sufficiently pure (uneven pigmentation or presence of non-pigmented cells), the cells are serially passaged for a further 1–2 passage cycles. Serial passaging increases RPE purity, however, with each passage, the cell culture cost and time increase as RPE cells undergo a cycle of dedifferentiation, expansion, and re-differentiation that takes about 4–6 weeks to be completed. Repeated passaging may cause RPE cells to lose their differentiation potential, resulting in non-pigmented, fibroblast-like cells that are unable to regain RPE identity [19].

Non-RPE cell contaminants prevent reliable analysis of RPE biology in vitro, and more importantly, the presence of even a few stem/progenitors cells can lead to uncontrolled proliferation and teratoma formation once transplanted in vivo, making the RPE monolayer unsuitable for clinical use [20, 21]. To ensure that these cells are ready for clinical application, subsequent labor-intensive methods are required to ensure removal of residual stem cells [22].

Each new protocol over the years has looked to improve the efficiency and speed with which RPE cells are produced [13, 14]. However, even the best of these protocols faces one of two significant problems. Firstly, reliance on some form of manual selection of pigmented colonies/embryoid bodies to improve RPE yield, which makes the process laborious and time consuming. Secondly, use of growth factors, cytokines, and small molecules to direct or hasten differentiation, which adds significantly to the cost of the process [23]. In addition, most current protocols do not address the problem of residual, non-RPE contaminants.

In this paper, we describe a novel protocol we have developed called the RPE PLUS protocol (RPE Purification by Lipoprotein Uptake-based Sorting) in an attempt to address all of the problems outlined above. Our protocol makes use of a simplified 2D induction method that obviates the need for manual selection of pigmented 3D structures during the expansion stage. We also demonstrate that RPE differentiation can be achieved in the absence of most previously described growth factors which significantly reduces the cost of production. Finally, we introduce a unique FACS-based, positive selection method for functional RPE (based on their ability to uptake lipoproteins), which ensures absence of non-RPE contaminants in the final culture. With this protocol, we see pigmentation as early as 21–23 days in the induction stage, and obtain functional, contaminant-free RPE cells after a single passage on transwell membrane supports.

## Methods

### Maintenance of human ESCs and iPSCs

H1 and H9 embryonic stem cells were purchased from WiCell Research Institute Inc. The hiPSC line GM23280A was obtained from Coriell Cell Repositories. Cells were cultured in chemically defined mTeSR medium (STEMCELL Technologies) on ESC-qualified Matrigel-coated (Corning) or CellAdhere™ Laminin-521-coated (STEMCELL Technologies) 6-well culture plates following manufacturer instructions.

Cells were passaged every 5–6 days using ReLeSR (STEMCELL Technologies).

### hESC and hiPSC induction to RPE fate

Overconfluent hESC or hiPSC cultures (day 0) were cultured for 7 days in neural induction medium which consisted of 50% DMEM/F12 + GultaMAX™ (GIBCO), 50% neurobasal medium (GIBCO), 0.5× B27 supplement (GIBCO), 0.5× N2 supplement (GIBCO), 55 μM 2-mercaptoethanol (GIBCO), and 1× l-glutamine (GIBCO). Medium was changed every 3 days. At day 7, cells were washed twice in DPBS no calcium and no magnesium (GIBCO) and cultured in RPE medium + 50 ng/ml recombinant human Activin A (PeproTech) until first signs of pigmentation were visible at the naked eye (normally at days 18–21). RPE medium is formulated as follows: DMEM, high glucose, GlutaMAX™ Supplement (GIBCO), 10% KnockOut Serum Replacement (GIBCO), 1× l-glutamine (GIBCO), 1× MEM Non-essential Amino Acid Solution (Sigma-Aldrich), 55 μM 2-mercaptoethanol (GIBCO), 1× penicillin-streptomycin (GIBCO). Medium was changed every 3 days. As soon as pigmentation started, cells were cultured in RPE medium without Activin A and the culture was carried on until at least 35–40% of the culture plate surface showed pigmentation (normally around day 35).

### RPE differentiation on transwell membranes

Transwell membranes of the desired size with 0.4 μm Pore Polyester Membrane Insert (Corning) were prepared 1 day in advance of the RPE differentiation step. The transwell membrane inserts were coated with 2 μg/ml CellAdhere™ Laminin-521 (STEMCELL Technologies) for 2 h at 37 °C and allowed to equilibrate overnight in RPE medium. At the end of the RPE induction step (day 35), samples showing at least 35–40% pigmented area were chosen for the RPE differentiation step. Samples were washed twice in 2 ml DPBS no calcium and no magnesium (GIBCO) and were divided into smaller pieces with the aid of a pipette tip. When the cell clusters were small enough to be resuspended with a P1000 pipettes tip, the samples were moved to a 15-ml falcon tube and centrifuged for 5 min at 300*g*. The pellet was then resuspended in Accutase (STEMCELLS Technologies) and incubated for 10 min at 37 °C. This cycle was repeated until a single-cell suspension was obtained. The single-cell suspension was then filtered through a 40-μm cell strainer (Falcon), centrifuged (5 min at 300 g), washed in DPBS and centrifuged again (5 min at 300*g*). Cells were finally counted and plated at a concentration of 500,000 cells/cm^2^ in RPE medium 10% KO serum. The day after seeding, the samples were washed thoroughly in order to remove debris/dead cells and provide a smooth surface for the adherent cells to grow. RPE medium supplemented with 10% KO serum was used for the first 3 days to help cell attachment and growth. After the first 3 days, cells were cultured in RPE medium 5% KO serum. Medium was changed every 3 days.

### Immunocytochemistry on cyst cryosections

hESC-derived cysts were manually picked at days 11–12, resuspended gently in PBS to free them from surrounding cells, and placed in a 15-ml falcon tube. Cysts were spun down for 1 min at 100*g*, fixed in 4% fresh PFA for 15 min at room temperature, washed in DPBS, and equilibrated in 30% sucrose solution overnight. The following day, cysts were frozen in Tissue-Tek O.C.T._TM_ Compound (Sakura). Cryosections of 10-μm thickness were mounted on Poly-l-lysine-coated microscope slides (Tekdon), washed in DPBS, and incubated with blocking buffer (DPBS, 1% BSA, 100 mM Glycine, 0.3% Triton X-100) for 30 min. Immunostaining was performed in antibody dilution buffer (DPBS, 0.5% BSA, 0.3% Triton X-100) overnight at 4 °C. The primary antibodies and their working dilutions were as follows: mouse anti-Pax6 (MAB5552 Millipore) 1:50, rabbit anti-ZO1 (402200 Thermo Fisher SCIENTIFIC) 1:200, rabbit anti-Rax (ab23340 Abcam) 1:200. Secondary antibodies conjugated with Alexa Fluor (Invitrogen) were diluted 1:1000 in blocking buffer and incubated for 45 min at room temperature. Slides were counterstained using VECTASHIELD® Antifade Mounting Medium with DAPI. Confocal images were taken using a Zeiss LSM 510 upright confocal microscope using a Plan-Apochromat *20x*/0.8 objective. DAPI, FITC, and Alexa-568 fluorophores were excited with a 405-nm (25 mW), 488-nm (30 mW), and 543-nm (1 mW) laser line and collected with a DAPI, FITC, and TRITC filter set respectively. Image acquisition and processing were performed in AxioVision software (Zeiss).

### Photoreceptor outer segment (POS) isolation and phagocytosis assay

POS were isolated from rat eyes as described by Schraermeyer et al. [24] with few modifications. Briefly, the anterior half of the eye was dissected, and the vitreous and retina were removed. Isolated retinae were agitated in KCl buffer (0.3 M KCl, 10 mM HEPES, 0.5 mM CaCl2, 1 mM MgCl2, 48% sucrose) at pH 7 and centrifuged at 7000 rpm in a table centrifuge (Sigma 3 K10) for 5 min. The supernatant containing POS was filtered through gauze, diluted with KCl buffer [1] with PBS and centrifuged at 5000 rpm for 7 min. To label POS with FITC, POS were suspended in a solution containing 10% sucrose and 20 mM sodium phosphate and incubated at 4 °C overnight with FITC isomer 1 (0.4 mg/ml, F-1907, Thermo Fisher SCIENTIFIC). After incubation, POS were centrifuged at 7000 rpm and aliquoted for storage at − 80 °C. For use in the phagocytosis assay, outer segments were thawed and centrifuged at 7000 rpm for 5 min and the supernatant aspirated. The outer segment pellet was resuspended in DMEM supplemented with 1% NEAA and 1% FBS. RPE cells on transwell membranes were incubated with outer segments (~ 10 POS/cell) prepared in this manner for 6 h at 37 °C. After 6 h, samples were thoroughly washed to exclude all POS that might be bound to the surface but not internalized by the RPE cells. The transwell permeable membranes were then removed from the transwell inserts using a scalpel and placed onto a microscope slide for imaging. Images were acquired using a Zeiss LSM 510 upright confocal microscope using a Plan-Apochromat *40x*/1.4 Oil DIC M27 objective. FITC and Alexa-568 fluorophores were excited with a 488-nm (30 mW) and 543-nm (1 mW) laser line and collected with a FITC and TRITC filter set. Image acquisition and processing were performed in AxioVision software (Zeiss).

### AcLDL uptake assay

RPE samples were incubated with 10 μg/ml Dil-conjugated AcLDL (Molecular Probes). Dil-AcLDL was added only to the upper chamber of the transwell membrane insert. After 2, 4, 6, and 12 h, samples were washed in DPBS to remove any excess Dil-AcLDL still present in the medium. RPE monolayers or monolayer fragments were gently detached from the transwell membrane with the aid of a P10 pipette tip. RPE monolayers or monolayer fragments were mounted on Poly-l-lysine-coated microscope slides (Tekdon) prior to imaging.

To look for evidence of POS phagocytosis in Dil-AcLDL-positive cells, hESC-derived RPE were first subjected to a 12-h incubation with medium containing Dil-AcLDL followed by a DPBS wash and an 8-h incubation with POS. After completion of incubation with POS, cells were washed to release any surface-bound POS. Images were acquired using a Zeiss LSM 510 upright confocal microscope using a Plan-Apochromat *10x*/0.45 or *20x*/0.8 objective. FITC and Alexa-568 fluorophores were excited with a 488-nm (30 mW) and 543-nm (1 mW) laser line and collected with a FITC and TRITC filter set. Image acquisition and processing were performed in AxioVision software (Zeiss).

### RT-qPCR analyses

Total RNA was extracted using a RNeasy kit (Qiagen), treated with RNase-free DNase I (Invitrogen) and reverse-transcribed with SuperScript II (Invitrogen). The resulting cDNAs were amplified with gene-specific primers using PowerUp™ SYBR™ Green Master Mix (Applied Biosystems™) following manufacturer recommendations. qPCR primer pairs were purchased from OriGene. Detailed list of the qPCR primers used is available in the additional files (Additional file 1: Table S1). Data are presented as relative expression of target gene to the mean of three housekeeping genes (GAPDH, HMBS, GPI) following the ΔCt method.

### Electron microscopy analyses

Cells were fixed in 2% glutaraldehyde and 2% PFA in 50 mM HEPES overnight at 4 °C. After several washes in 100 mM HEPES and PBS, they were post-fixed with 1% OsO4/PBS for 2 h on ice, washed with PBS and water, and en bloc contrasted with 1% uranyl acetate in water followed by a further 2 h on ice. Samples were dehydrated in graded ethanol, infiltrated in ethanol/epon mixtures (1:3, 1:1, 3:1) followed by pure epon, and cured at 60 °C for 24 h. Samples were then cut to small pieces (1–2 mm), remounted for cross-sectioning of the RPE layer on empty epon dummy blocks, and cured for another 24 h at 60 °C. Ultrathin sections were stained with lead citrate and uranyl acetate, and visualized with a transmission electron microscope (JEM-1400, JEOL).

### Enzyme-linked immunosorbent assay (ELISA) for vascular endothelial growth factor (VEGF)

The culture medium from confluent RPE samples was collected 24 h after the medium was changed. Secretion levels were measured by the human VEGF-ELISA Kit (eBioscience) following the manufacturer’s instructions.

### Measurement of transepithelial electrical resistance (TEER)

TEER of RPE cells on transwell membranes was measured using an EVOM3 epithelial voltohmmeter (World Precision Instruments, Hamden, CT) with chopstick electrodes. To obtain TEER values of a target sample, the voltohmmeter output value of the blank was subtracted from the sample output and multiplied by the surface area of the transwell membrane. TEER = (R sample − R blank) × effective surface area.

### Fluorescence-activated cell sorting of Dil-AcLDL positive cells

RPE samples were labeled with Dil-AcLDL overnight, washed twice in DPBS, and dissociated by Accutase (STEMCELLS Technologies) incubation for 20 min at 37 °C. After 10 min, cells were resuspended thoroughly to help dissociation. Once single-cell suspension was achieved, cells were filtered through a 40-μm cell strainer, centrifuged (5 min at 300*g*), washed in DPBS, and centrifuged again (5 min at 300*g*). Cells were sorted using BD FACSAriaIII (Becton Dickinson). Non-stained RPE cells were used as negative control.

### Statistical analysis

Data are presented as mean ± standard error of the mean (SEM). For qPCR data, expression of each gene was analyzed separately with its own control to prevent influence of multiple comparisons. In addition, where indicated, additional analysis was performed to generate Cumming plots to show individual data points and effect size. Due to space constraints in the main figures, all Cumming plots for the relevant main figures have been included in the additional files. Statistics were carried out on a minimum of three independent experiments.

## Results

**Fig. 1:**
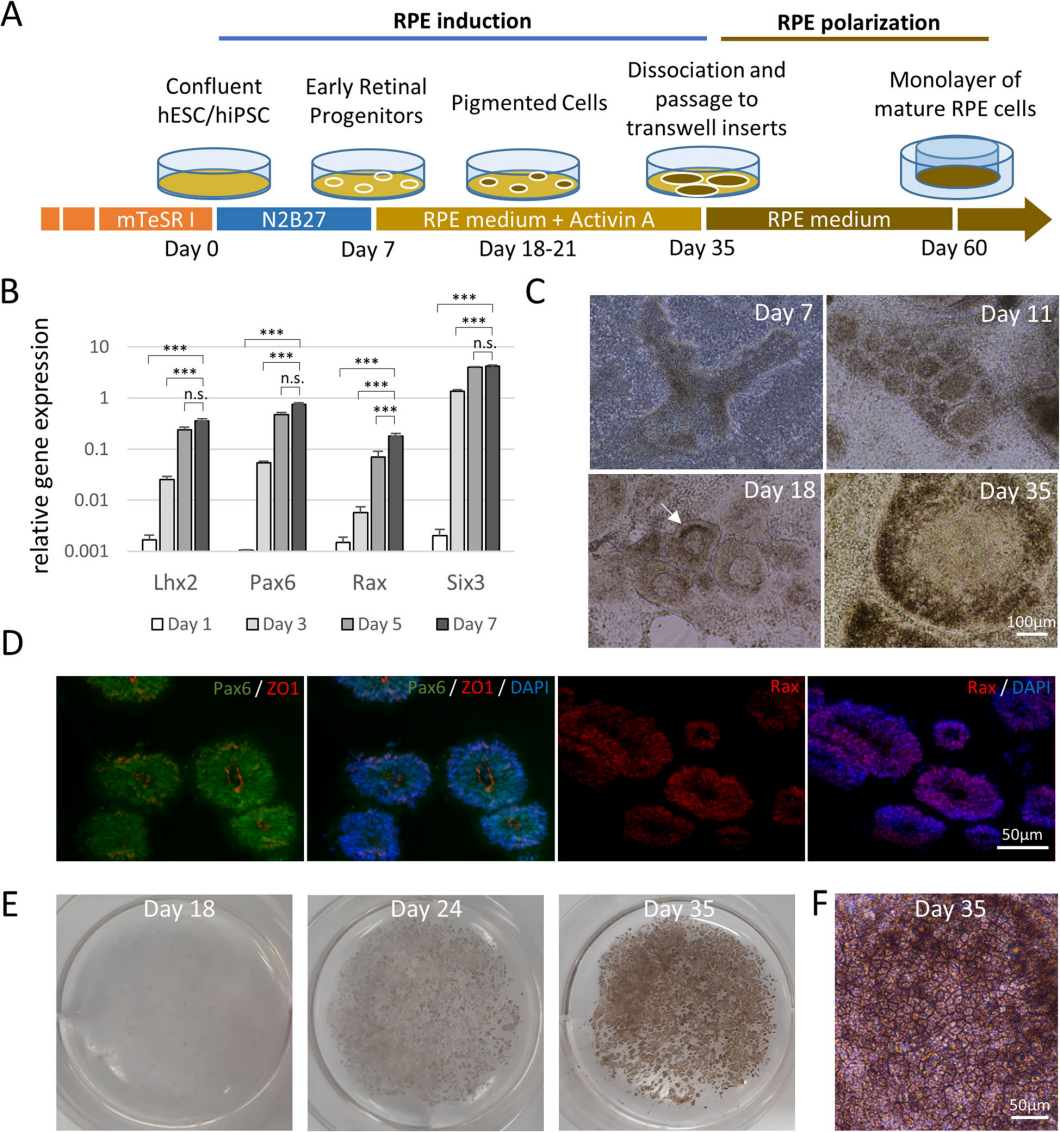
Rapid and efficient differentiation of hESC/hiPSC-derived RPE cells. **a** Schematic of differentiation protocol. **b** RT-qPCR gene expression analysis of genes involved in early retinal identity. Data presented as target gene expression relative to mean expression of three housekeeping genes (*n* = 5). Data expressed as mean ± SEM. Data are represented as mean ± SEM; *p < 0.05, **p < 0.01, ***p < 0.001, n.s. = not significant (Student *t* test). **c** Representative brightfield microscopy images of H1 hESC confluent cell culture undergoing differentiation to RPE. Arrow indicates first sign of pigmentation. **d** Immunocytochemical analysis of cryosections of hESC-derived cysts at day 18. Ten-micrometer-thin slices were stained for PAX6, ZO1, and RAX and nuclei were counterstained with DAPI. **e** Representative images of H1 hESC culture developing pigmentation over time in a six well plate. **f** High magnification brightfield image of RPE cells at the end of the induction stage

Figure 1a summarizes the two-step protocol (induction and polarization), we used to differentiate hESC/hiPSC into mature and functional RPE in vitro. The stage of induction directs pluripotent stem cells towards a retinal progenitor fate followed by early RPE differentiation. The stage of polarization subsequently allows the newly formed RPE to acquire mature morphology and become functional.

### Human ES or iPS cells can be induced to RPE in simplified 2D culture

Once pluripotent cells reach confluence, they are switched from stem cell culture medium to neural induction medium at day 0 of the culture. The cells are maintained in the neural induction medium for 7 days with media changed every 3 days. We find that this simple induction technique is sufficient to enable upregulation of the eye-field genes *LHX2*, *PAX6*, *RAX*, and *SIX3* and guide the pluripotent stem cells through an early retinal fate in just 5–7 days (Fig. 1b, for raw data point distribution and effect size see Additional file 2: Figure S1).

At day 7, the cells are switched to and maintained in RPE medium supplemented with human Activin A. Note that the cells remain in 2D culture in the same culture plate throughout this process. Starting from days 11–12, self-organizing donut-shaped structures begin to appear within the culture, particularly in areas of over-confluence (Fig. 1c). Immunofluorescence revealed that these structures demonstrate robust expression of early retinal markers PAX6 and RAX, as well as the neuroepithelial marker ZO1 (Fig. 1d). We believe these structures are comparable to the neuroepithelial cysts described previously by Zhu et al. in their 3D protocol for RPE differentiation [16].

With time in culture, these flattened neuroepithelial cysts become increasingly evident and numerous. Eventually they grow beyond the original cystic structures and begin to generate pigmented cells at their edges around days 21–23. (Fig. 1c). Such pigmented areas are visible by the naked eye (Fig. 1e). Once this is seen, Activin A is withdrawn from the medium and the cells are maintained in RPE medium without Activin A until days 30–35 to allow pigmented cells to expand and cover at least the 35–40% of the total surface (Fig. 1e). At this stage, microscopically, the pigmented cells demonstrate the typical cobblestone morphology of mature human RPE cells (Fig. 1f).

This induction protocol was tested on two different hESC lines (H1 and H9) as well as iPSC lines and found to be equally effective. Importantly, in the case of the hiPSC cells, culture of stem cells on the traditional Matrigel matrix or the xeno-free laminin 521 substrate produces equivalent results, (Additional file 3: Figure S2) suggesting that this protocol could also be adapted for xeno-free clinical applications.

To further simplify the process, we also evaluated the effect of reducing/removing Activin A from the culture. Reduction or removal of Activin from the RPE induction medium does not affect the efficiency of the RPE induction process. However, the time required for pigmentation to be achieved changes with varying concentrations of Activin. Samples with 100 ng/ml Activin A became pigmented at day 21, while those incubated with 50 ng/ml Activin A showed first signs of pigmentation at day 23. In the absence of Activin A, pigmentation was first noticed around day 30 (Additional file 3: Figure S2). However, by day 40, samples cultured without Activin A also reach pigmentation levels equivalent to those cultured in 50 or 100 ng/ml of Activin. This suggests that RPE differentiation could be achieved in the absence of Activin A if the user was willing to culture the cells for a further 10–15 days (data not shown).

At the end of the induction phase, large numbers of contaminant non-pigmented cells are present in the culture. Further purification and functional maturation of the RPE cells is achieved through the polarization step achieved by culture on permeable membranes.

### Post induction single passage onto transwell membranes forms polarized RPE monolayers

Once 35–40% of the well area is covered by pigmented cells (usually around days 30–35), the cells are plated on transwell permeable membranes to promote RPE maturation. The transwell membranes support growth of epithelial cells in a uniform monolayer, promoting formation of tight junctions as well as acquisition of polarity, which is critical to RPE cell function. Further, culture on the transwell membranes enables functional testing of transepithelial electrical resistance (TEER) and polarized secretion of cytokines to verify the quality of the RPE monolayer. The passage also allows selective culture of RPE cells thereby aiding in some degree to removal of non-RPE contaminants.

**Fig. 2:**
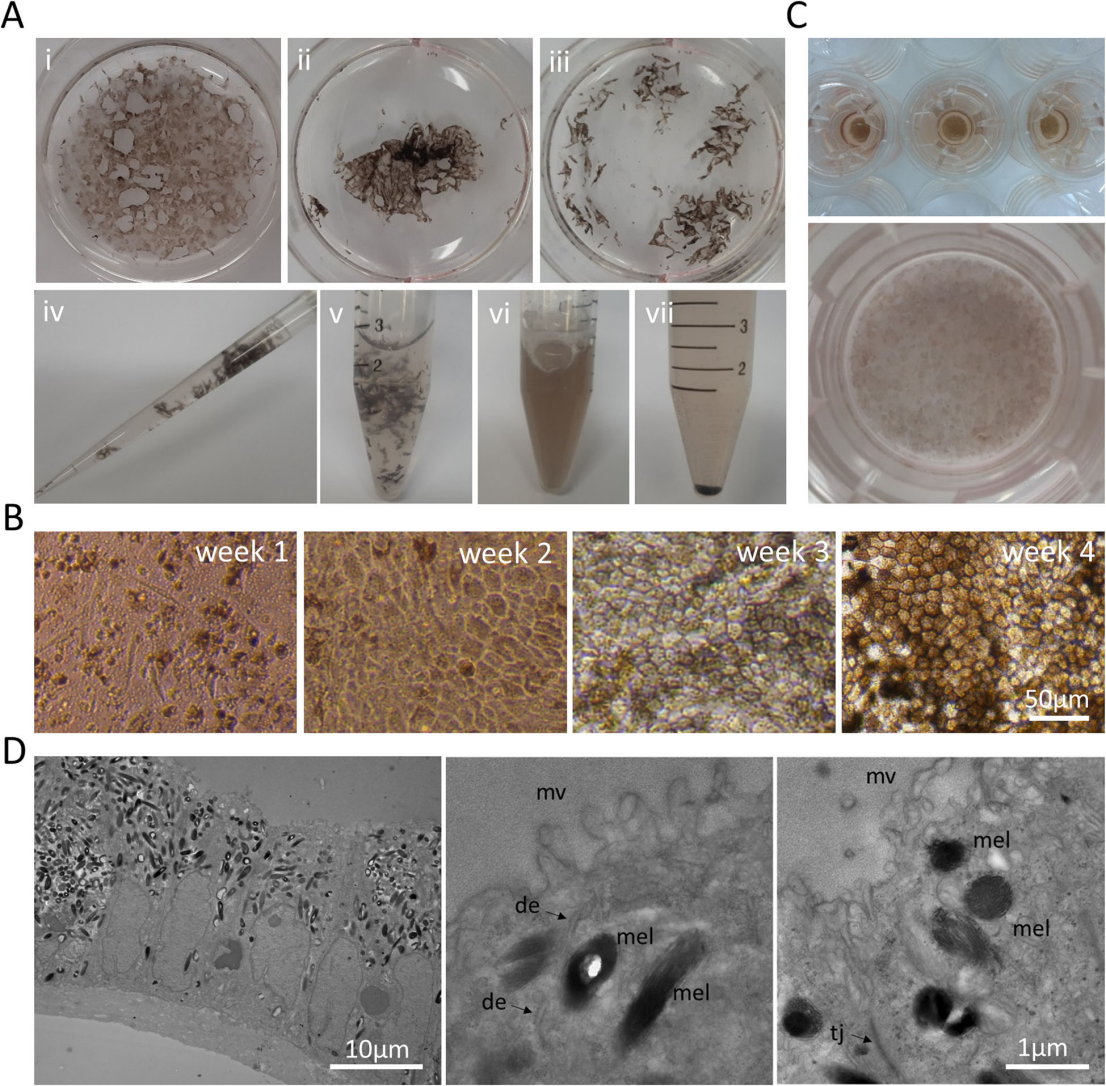
Expansion and differentiation of polarized RPE cells on transwell membranes. **a** Dissociation of RPE cells obtained after the RPE induction step into single-cell suspension. (i) Samples were washed twice in DPBS and resuspended in Accutase, (ii) detached from the culture plate surface, and (iii) broken into small pieces using pipette tips. (iv) Small clusters of samples were resuspended thoroughly using a P1000 pipette, (v) transferred to a 15-ml Falcon tube, and incubated for 10 min at 37 °C. (vi) Samples were pipetted vigorously and incubated for other 10 min at 37 °C, resuspended several times, filtered through a 40-μm cell strainer, and (vii) centrifuged to recover the cellular pellet. **b** Representative images of RPE cells during expansion and differentiation on transwell membranes. The seeded cells adhere to the transwell membrane surface and start dividing (week 1). Cells expand until confluence is reached (week 2). RPE progenitors begin to assume the typical RPE morphology and pigmentation level (week 3). Mature RPE cells display highly pigmented cobblestone-like morphology (week 4). **c** Representative images of 6.5 mm diameter (upper panel) and 24 mm diameter (lower panel) transwell membranes after 4 weeks of culture. **d** Transmission electron microscopy images of highly pigmented samples highlighting cellular organization into a monolayer of tightly connected columnar-shaped cells with nuclei on the basal side and melanosomes on the apical side (left panel) and details of RPE cell ultrastructure. de = desmosome, mel = melanosome, mv = microvilli, tj = tight junction

In order to passage, the cells first need to be dissociated into a single-cell suspension. Due to large amounts of extracellular matrix proteins produced by these cells in culture during induction, vigorous dissociation is required to obtain a single-cell suspension. The cell layers dissociate easily from the culture plate, but require further trituration using pipette tips to break them into smaller pieces. (Fig. 2a i-iii). Once the clusters are small enough to be resuspended (Fig. 2a iv), the cells are incubated for 10 min in Accutase at 37 °C followed by vigorous pipetting. The cycle can be repeated further for 5–10 min to ensure formation of single-cell suspensions (Fig. 2a v, vi). The suspension is then filtered through a 40-μm cell strainer and cells centrifuged to obtain a cell pellet (Fig. 2a vii). Cells are then seeded onto transwell membranes coated with Laminin 521 or Growth Factor Reduced Matrigel at a concentration of 500,000 cells/cm^2^ in RPE medium. The following day, a variable percentage of the seeded cells (ranging from 10 to 40%) attaches to the transwell membrane surface, grows to confluence, and undergoes a cycle of dedifferentiation, expansion, and re-differentiation [13, 19, 25] to once again progressively assume the typical RPE cobblestone-like morphology with increasing levels of pigmentation (Fig. 2b,c). This process takes approximately 30–40 days. The RPE thus achieved are fully polarized as evidenced by their appearance on transmission electron microscopy that highlights the RPE cell ultrastructure, their columnar shape, and the apical microvilli (Fig. 2d).

### hESC-derived RPE demonstrate VEGF production, high TEER, and POS phagocytosis in vitro

**Fig. 3:**
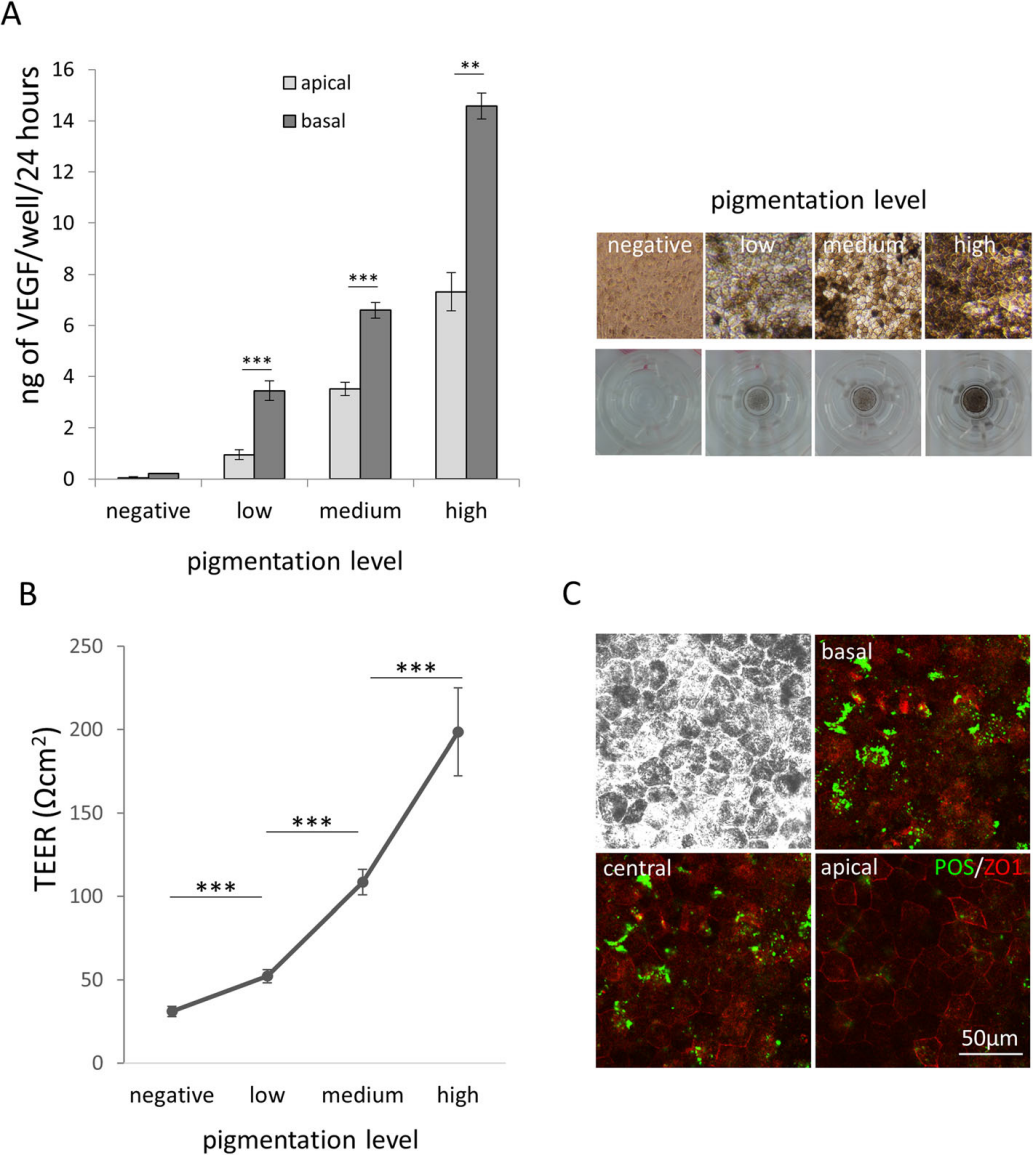
Functional assessment of hiPSC-RPE cell sheets in vitro. **a** ELISA of VEGF secretion by hESC-RPE (H1) on the apical and basal side of a 6.5-mm transwell membrane over a period of 24 h (VEGF: negative apical 0.05 ± 0.04, negative basal 0.21 ± 0.01, low apical 0.95 ± 0.19, low basal 3.44 ± 0.38, medium apical 3.51 ± 0.26, medium basal 6.59 ± 0.26, high basal 7.31 ± 0.74, high basal 14.58 ± 0.51 ng/well/24 h) (*n* = 5). Right panels show images of hESC-RPE exhibiting different grades of pigmentation as reference. **b** TEER of hESC sheets on transwell membranes during RPE differentiation (*n* = 5). **c** Confocal images taken at different depths (z-stack) of RPE cells challenged with FITC-conjugated rat POS for 6 h. Brightfield (upper left) and fluorescence images at different focal planes (basal, central, apical) show internalization of POS (green) in the cytoplasm of RPE cells. ZO1 staining (red) identify tight junctions expressed on the apical side. All pooled data are represented as mean ± SEM; *p < 0.05, **p < 0.01, ***p < 0.001 [Student *t* test]

hESC-derived RPE cells express VEGF in a polarized fashion similar to in vivo RPE where this function supports growth and homeostasis of the choriocapillaris in contact with the basal surface of the RPE monolayer [26]. ELISA analysis of media from the basal and apical sides of the transwell membranes on which the RPE were cultured showed polarized expression of VEGF and that the level of expression increases with the level of pigmentation (Fig. 3a, for raw data point distribution and effect size see Additional file 4: Figure S3).

Furthermore, RPE cultured in this manner show formation of strong tight junctions, similar to in vivo RPE, as evidenced by high levels TEER in vitro. TEER values increase over time correlating with the levels of pigmentation, reaching average levels of 200 Ωcm^2^ at 8 weeks after replating on transwell membranes (Fig. 3b, for raw data point distribution and effect size see Additional file 5: Figure S4).

Finally, RPE cells cultured in this manner demonstrate the ability to phagocytose photoreceptor outer segments (POS), a function that, in vivo, is critical for the maintenance of the visual cycle and photoreceptor homeostasis [27, 28]. We challenged RPE cells by incubating them with FITC labeled rat POS for 6 h (approximately 10 POS/cell). Subsequent washes to remove non-internalized POS, immunostaining, and confocal microscopy showed absence of POS at the apical surface (identified by apical ZO1 staining) and presence of POS at the basal and central focal plane of the RPE cells, thus confirming that the POS fragments were not simply present at the cell surface, but were indeed internalized by the RPE (Fig. 3c).

### Mature RPE cells express high levels of lipoprotein receptors and efficiently internalize AcLDL

Although we were able to achieve high-quality functional RPE, the protocol as such still did not allow for complete removal of non-RPE contaminants. To address this, we turned to the lipoprotein uptake ability of RPE as a possible solution.

**Fig. 4:**
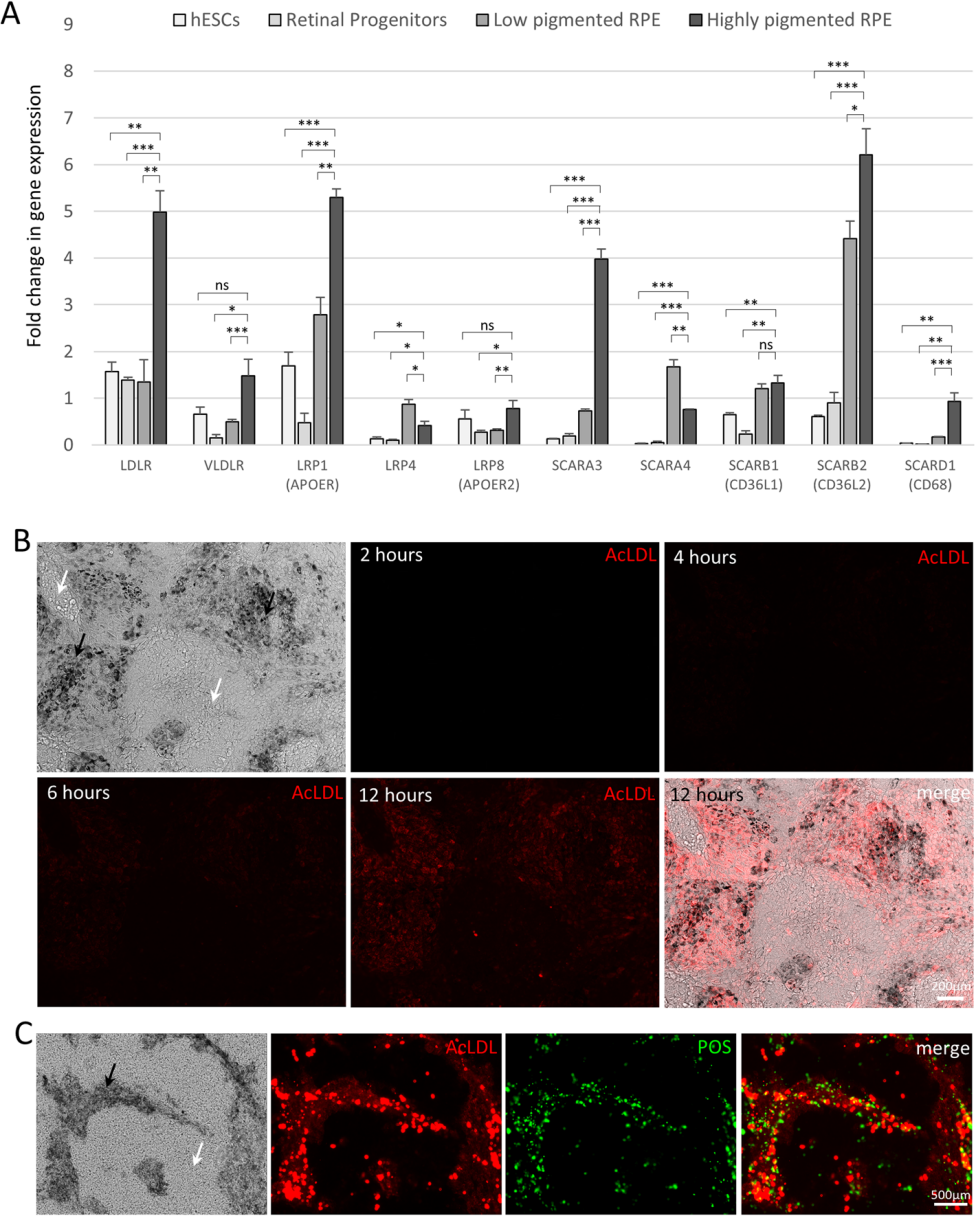
Overexpression of lipoprotein receptors in RPE cells allows for selective labeling of mature RPE cells by Dil-AcLDL uptake. **a** RT-qPCR analysis of gene expression in stem cells (day 0), retinal progenitors (day 7), low pigmentation RPE cells (~ day 45), and high pigmentation RPE cells (~ day 70). Gene expression results are represented as fold change relative to the geometric mean of three housekeeping genes: GAPDH, GPI, HMBS (*n* = 3). All pooled data represented as mean ± SEM; *p < 0.05, **p < 0.01, ***p < 0.001 [Student *t* test]. **b** Dil-AcLDL uptake assay. Fluorescence microscopy images of low-quality RPE monolayers challenged with Dil-AcLDL (red) for 2, 4, 6, and 12 h. **c** Colocalization of signals from Dil-AcLDL (red) uptake assay (overnight incubation) and FITC-POS (green) phagocytosis assay (6-h incubation). Black arrow: pigmented RPE cells. White arrow: non-RPE cells

It is known that human RPE cells express several different lipoprotein receptors [29–31]. We analyzed our stem cell-derived RPE at different stages of differentiation (D0, D7, polarized cells with low pigmentation at D50, polarized RPE with high pigmentation at D70) and found that *LDLR*, *VLDLR*, *LRP1*, *SCARA3*, *SCARB2,* and *SCARD1* mRNA expression was strongly upregulated in differentiated RPE cells compared to retinal progenitors and stem cells (Fig. 4a, for raw data point distribution and effect size see Additional file 6: Figure S5). This particular panel of lipoprotein receptors appeared to correlate with increasing maturity of the RPE in vitro. A full list of lipoprotein receptors tested is available in Additional file 7: Figure S6.

The scavenger receptors (in particular SCARA3 and SCARB2) overexpressed in our stem cell-derived mature RPE have been shown to have high affinity for acetylated low-density lipoproteins (Ac-LDLs) [32]. Based on this, we decided to test the ability of our hESC-derived RPE cells to uptake Dil-labeled acetylated LDL (Dil-AcLDL). We hypothesized that due to their higher expression of scavenger receptors, mature hESC-derived RPE cells would possess a selective ability to efficiently uptake Dil-AcLDL, an activity that would be absent in the non-RPE or poorly differentiated RPE cells. If the hypothesis was true, Dil-AcLDL could be used to selectively isolate functional RPE from contaminant cells by fluorescence-activated cell sorting (FACS).

Accordingly, our hESC-derived RPE cells were incubated with Dil-AcLDL for 12 h and Dil-AcLDL uptake monitored by fluorescent microscopy over time. The test was performed on poorly differentiated samples (with less than 70% cells showing typical RPE morphology on transwell membranes) which allowed for internal comparison of differentiated and undifferentiated cells. We found that only cells displaying RPE morphology and pigmentation were able to uptake Dil-AcLDL. Weak Dil-AcLDL uptake was visible 6 h post incubation and increased significantly by 12 h post incubation (Fig. 4b). The pigmented cells which demonstrated Dil-AcLDL uptake were further challenged with fresh medium containing FITC-conjugated POS to assess their RPE function. The pigmented cells showed excellent colocalization of pigmentation, Dil-AcLDL, and FITC-POS (Fig. 4c). This confirmed that pigmented cells which showed robust Dil-AcLDL uptake were indeed functional and metabolically active RPE.

### FACS-based selective subculture of Dil-AcLDL-positive RPE cells generates contaminant-free RPE monolayers

**Fig. 5:**
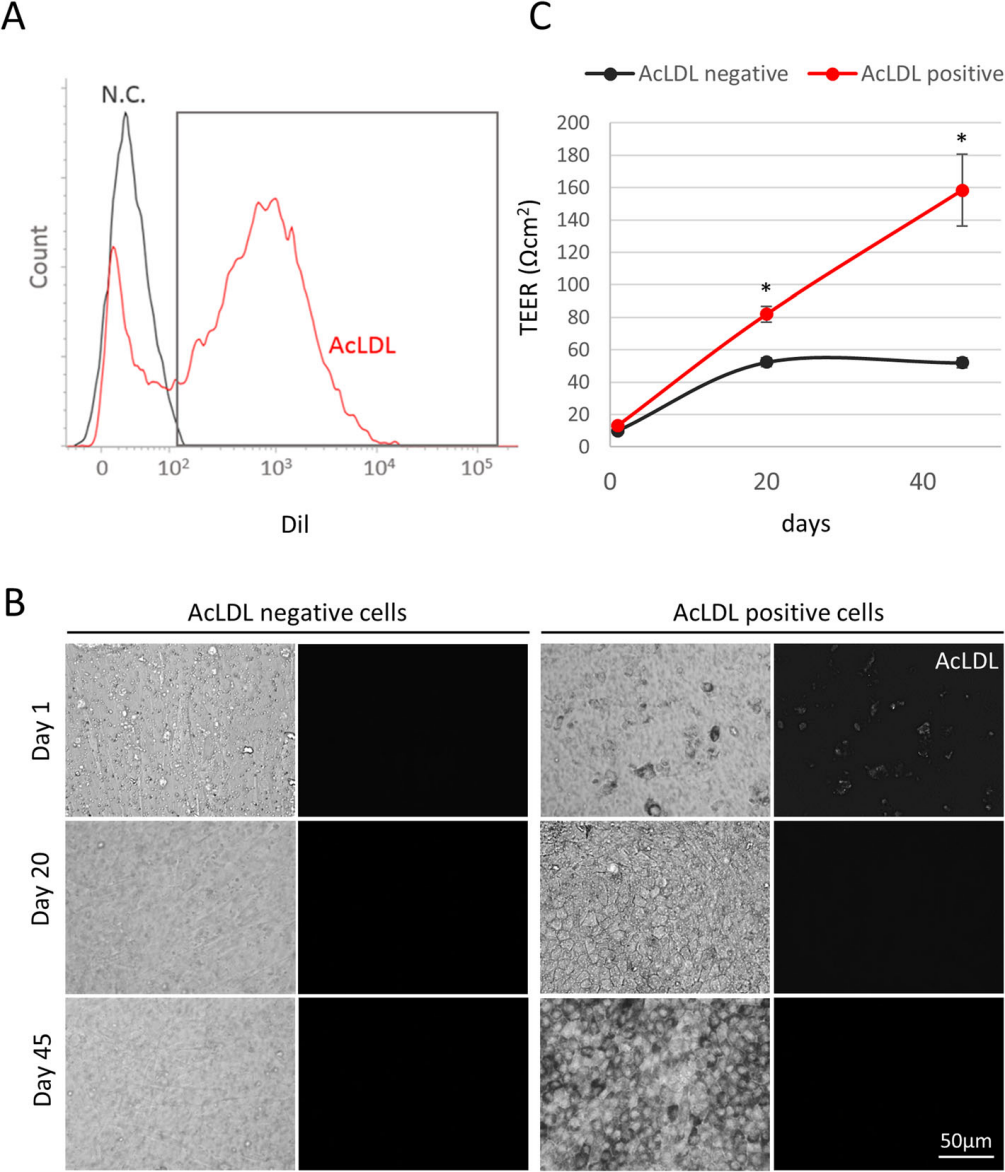
Purification of AcLDL positive cells by cytofluorimetry allows the generation of highly pure RPE monolayers. **a** Representative image of cell sorting based on Dil-AcLDL labeling. RPE samples were incubated overnight with 10 μg/ml Dil-AcLDL. Cells were washed in DPBS, dissociated by Accutase treatment and filtered through a 40-μm cell strainer prior to cell sorting. Cells were sorted based on staining by Dil-AcLDL (red). Unlabeled cells from RPE samples were used as negative control (N.C.). **b** AcLDL-negative (left panel) and AcLDL-positive (right panel) cells were collected after sorting and seeded at a concentration of 10^5^ cells/cm^2^ in transwell filters coated with growth factor reduced Matrigel. Cell morphology and persistence of AcLDL molecules in AcLDL-positive cells were assessed by brightfield and fluorescent microscopy, respectively, at days 1, 20, and 45. **c** TEER values were measured at days 1, 20, and 45 using an EVOM2 voltohmmeter. Data are expressed as mean ± SEM. *P < 0.05 [Student *t* test]. *n* = 3

Having demonstrated that only mature functional RPE selectively uptake Dil-AcLDL, we tested the ability to obtain pure RPE cell monolayers by sorting Dil-AcLDL-positive cell populations by FACS. hESC-derived RPE samples rich in contaminant cells were simply incubated with Dil-AcLDL overnight and dissociated into a single cell suspension before cell sorting (Fig. 5a). The Dil-AcLDL negative and positive populations were plated separately onto new transwell membranes at a concentration of 10^5^ cells/cm^2^ and maintained in culture as before. Over time, Dil-AcLDL-negative cells failed to differentiate into RPE cells, generating confluent cultures of non-pigmented, large, fibroblast-like cells (Fig. 5b). By contrast, AcLDL-positive cells were able to go through the previously described cycle of dedifferentiation (first 3–4 days of culture), expansion (reaching confluence at day 20), and re-differentiation (increasing levels of pigmentation and RPE cobblestone-like morphology from day 20 onward) to form a robust RPE monolayer. In the Dil-AcLDL-positive cells, the fluorescent signal was lost rapidly once the cells were replated with no signal detectable by day 20. (Fig. 5b). Importantly, Dil-AcLDL-positive cells established high levels of TEER during differentiation (158.3 ± 22 Ωcm^2^ at day 45) in contrast to their Dil-AcLDL-negative counterpart (52 ± 3 Ωcm^2^) (Fig. 5c, for raw data point distribution and effect size, see Additional file 8: Figure S7). This confirmed that Dil-AcLDL-based sorting was able to selectively isolate functional RPE while removing any non-RPE contaminants resulting in a pure functional RPE monolayer.

**Fig. 6:**
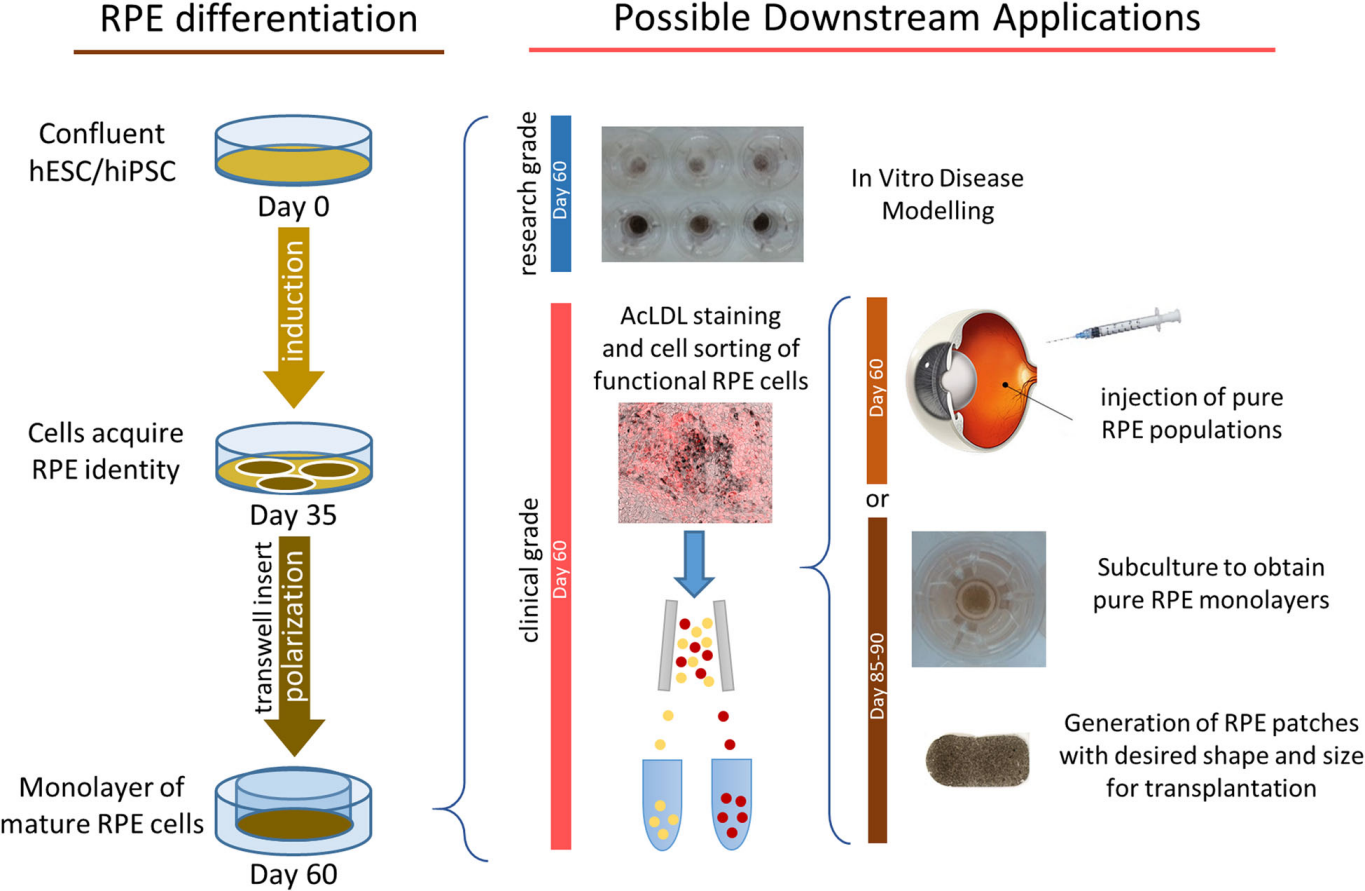
Schematic of the RPE PLUS protocol workflow and possible applications

Taken together, all these results validate our RPE PLUS protocol as a viable and convenient way to generate and purify functional, high-quality RPE cells to be used in research and clinical applications (Fig. 6).

## Discussion

The RPE PLUS protocol described here begins with a modification of the technology described by Zhu et al. in which neuroepithelial cysts expressing early retinal markers (*PAX6*, *RAX,* and *SIX3*) are generated with unprecedented speed (5 days) using a 3D culture [16]. However, setup of 3D cultures is manpower-intensive and technically challenging. To simplify this process, we exploit the ability of overconfluent 2D stem cell cultures to self-generate neuroepithelial cysts under pro-neuronal conditions. Not only does this make the process technically easier, it also obviates the need for reagents containing animal-derived components like Matrigel, which are critical to maintaining the speed of neural induction described by Zhu et al. We found that stem cells cultured on xeno-free laminin were able to produce similar results as those on Matrigel suggesting that this could be adapted to a xeno-free protocol. Incidentally, we also noticed that RPE cells can be generated adjacent to overconfluent areas within the 2D cultures, even in the absence of evident neuroepithelial cyst formation. This is possibly related to the ability of the cells to reorganize into multilayered structures during the neural-induction step in these overconfluent areas.

The neuronal induction step is followed by the RPE induction whereby cells are cultured in RPE medium supplemented with human Activin A only, contrary to all other protocols that propose the use of a cocktail of cytokines [12, 17, 18]. In presence of Activin A, pigmented patches of cells, indicative of RPE progenitors, appeared around days 18–21, consolidating and expanding over time. Strikingly, Activin A is not critical to the generation of pigmented cells. When using RPE medium without Activin A, pigmentation occurs at a slower pace but the final efficiency of RPE induction remains comparable to samples cultured in medium with Activin A. This suggests that Activin A accelerates rather than induces the RPE differentiation process, which likely occurs by default once the samples undergo the neuronal induction step [33]. Indeed, samples that did not undergo neural induction failed to generate pigmented cells after 40–50 days of culture even in Activin A supplemented RPE medium (data not shown). The major drawback of using recombinant Activin A for large-scale RPE production is the high cost, especially for the Good Manufacturing Practice (GMP) grade version of the product. Our protocol allows for generation of high-quality RPE cells without Activin A at the cost of prolonging the RPE induction step for 10–15 more days, compared to the workflow reported in Fig. 1a.

Since the RPE PLUS protocol is carried out in 2D, the amount of cell treatment and number of passages required during the differentiation process are reduced. The entire induction phase is carried out in the same culture dish without additional passage. By day 30–35 post induction, culture plates typically show 35–40% of pigmented surface area at which time the first passage occurs. The cells are dissociated into single cells and replated onto transwell membranes to induce expansion and polarization of RPE cells. Although cobblestone pigmented morphology may be seen on coated culture plates, functionality is not optimal in these conditions and the transition to transwell membranes allows maturation of the RPE monolayer with acquisition of all the features of functional human RPE (morphology, pigmentation, polarized VEGF expression, high TEER values, and ability to phagocytose POS) after 30–40 days of culture.

Persistence of any residual stem cells in biological samples destined for transplantation can lead to uncontrolled proliferation of stem cells and teratoma formation. In the first attempts at autologous transplantation of hiPSC-derived RPE cells in a patient with neovascular AMD, extensive transcriptomic and epigenetic profiling, as well as teratoma formation assay in immunodeficient mice, was performed to ensure safety prior to transplantation [6, 34]. In the absence of any other known methods of functional RPE purification, these expensive methods are the gold standard for safety and quality assessment and need to be performed in every case before stem cell-derived RPE can be used clinically.

To overcome this challenge, we propose a FACS-based method of RPE purification using the ability of functional RPE cells to uptake a carbocyanine dye conjugated AcLDL. Mature human RPE are known to express several different lipoprotein receptors that bind both native or modified (acetylated or oxidized) LDLs [30, 35, 36]. Similar expression of lipoprotein receptors is seen in RPE generated using our RPE PLUS protocol. We show that *LDLR*, *VLDLR*, *LRP1* (*APOER*), *SCARA3*, *SCARB2,* and *SCARD1* are significantly upregulated in mature RPE (and to a minor extent in differentiating immature RPE) but not in undifferentiated stem cells or early retinal progenitors. Upon challenging mixed differentiated and undifferentiated cells with fluorophore-labeled AcLDL (Dil-AcLDL), mature RPE demonstrates robust selective fluorophore uptake while the non-pigmented undifferentiated cells do not. Additionally, the cells that demonstrate AcLDL uptake also show robust internalization of FITC-conjugated POS confirming their functional RPE phenotype. FACS-based selective isolation and subculture of AcLDL-positive cells results in formation of new homogeneous RPE monolayers. On the other hand, AcLDL-negative cells form monolayers of non-pigmented, large, fibroblast-like cells.

We opted for AcLDL given the high affinity of AcLDL for scavenger receptors [30]. There have been other previous attempts at selective isolation of RPE cells in vitro. Choudhary et al. showed that CD59 expression allows discrimination of RPE cells from residual stem cells and that subculture of CD59-positive cells results in a uniform monolayer of polygonal-shaped cells [37]. However, it was not clear whether the subsequent monolayer demonstrated any hallmarks of RPE functionality such as pigmentation, acquisition of high TEER values, or phagocytic ability.

Incidentally, we did detect a small proportion of non-pigmented cells demonstrating AcLDL uptake within our cultures (Fig. 4b). Despite being non-pigmented, these cells showed a hexagonal morphology and are probably immature RPE cells. This postulate is supported by our transcriptional analysis of lipoprotein receptor expression in differentiating cells which suggests that immature RPE cells also express lipoprotein receptors, albeit at lower levels than that seen in mature RPE.

The RPE PLUS protocol also lends itself towards use in clinical applications. It can be adapted to xeno-free conditions, as Matrigel used in the protocol can be substituted by the xeno-free, chemically defined, CellAdhere™ Laminin-521. The carboycanine lipoprotein compound DiI-AcLDL used to label the cells for purification is undetectable in the RPE cells 48 h after labeling. Although the likelihood of any remaining dye in the cells used for transplantation after 45 days in culture is minimal, it is important to consider the safety implications of trace amounts of DiI-AcLDL being transplanted if these RPE cells are used clinically. Carbocyanine dyes on their own or coupled with lipoproteins have been used extensively in pre-clinical models, are gaining traction in the field of near infrared (NIR) theranostics for cancer, and have previously been used clinically for photodynamic therapy in cancer [38, 39]. DiI-labeled motoneurons have also been shown to remain viable up to 1 year in vivo [40, 41]. Exosomes and extracellular vesicles containing lipoproteins are being tested widely (over 90 exosome-based treatments are currently listed in www.clinicaltrials.gov) in diagnostic and therapeutic clinical trials [42, 43]. These studies set precedence and suggest that carbocyanine lipid labeling may be safe for clinical use. Demonstration of complete expulsion of DiI-AcLDL from cells prior to transplantation or targeted pre-clinical safety testing will add further support to the use of RPE cells derived using this RPE PLUS protocol for clinical use.

## Conclusions

In summary, the RPE PLUS protocol described here presents several advantages over current RPE culture and purification methods:

1. 2D culture reduces technical difficulty and obviates need for the animal-derived Matrigel components
2. Induction media and RPE media are cytokine scarce reducing culture costs
3. Passage onto transwell membranes for RPE maturation and polarization can be done by simply dissociating cells into a single-cell suspension. This removes the need for manual picking, presenting a tangible advantage for scalability.
4. The Dil-AcLDL-based purification is a significant advance in the RPE purification process: (a) the process is simple, requiring only overnight incubation of the cells Dil-AcLDL followed by dissociation into cell suspension, (b) there is no need for antibody staining thereby minimizing cell stress and maximizing cell viability after reseeding, (c) high-quality RPE cell suspensions or monolayer can be obtained immediately after cell sorting or after a single passage post sorting, respectively, (d) by selecting only cells that can uptake Dil-AcLDL, the protocol allows for simultaneous dual selection of only viable and functional RPE cells.
  Protocols for derivation of RPE from pluripotent stem cells need to be simple and scalable if the increasing demands for these cells in clinical and research applications are to be met in a cost-efficient manner. The RPE PLUS protocol we present here reduces the time, technical difficulty, and reagents needed to achieve RPE differentiation from stem cells in vitro. In addition, we introduce a method to purify mature and polarized RPE cells based on their ability to uptake AcLDL. This presents a significant advance in the technology for generation of large batches of high-quality, functional, RPE cells for regenerative medicine and AMD disease modeling.

## Supplementary information

Additional file 1: **Table S1.** List of qPCR primers.

Additional file 2: **Figure S1.** RT-qPCR gene expression analysis of genes involved in early retinal identity. The mean difference in expression of each gene at day 3, 5 and 7 of differentiation is compared against the shared control Day 1 and shown as Cumming estimation plots. The raw data is plotted on the upper axes. On the lower axes, mean differences are plotted as bootstrap sampling distributions. Each mean difference is depicted as a dot. Each 95% confidence interval is indicated by the ends of the vertical error bars.

Additional file 3: **Figure S2.** Differentiation of hIPSC into RPE cells under different experimental conditions. Representative image of Day 35 hiPSC line GM23280A differentiated to RPE in a 6-well plate under different experimental conditions. GM23280A cells were cultured on Matrigel-coated plates in mTesrI medium. The last passage prior to differentiation experiment was done in a plate coated with Matrigel or Laminin 521 following manufacturer recommendations. Cells were cultured in mTesrI until confluence and then switched to neuronal induction medium N2B27 for 7 days. After the 7 days neural induction cells were cultured in RPE medium supplemented with 0, 50, 100 ng/ml human Activin A. Pigmentation is indicative of RPE differentiation and maturation.

Additional file 4: **Figure S3.** Polarized VEGF secretion assay. ELISA VEGF secretion by hESCs-RPE (H1) on the apical and basal side of a 6.5 mm transwell insert over a period of 24 h. The apicobasal VEGF secretion for each of the 4 samples with varying levels of cell pigmentation are shown in the Cumming estimation plot.

Additional file 5: **Figure S4.** Trans Epithelial Electrical Resistance (TEER) assay. Assessment of TEER of hESCs sheets on transwell inserts during RPE differentiation. The comparison of TEER in increasingly pigmented cells against cells with no pigmentation are shown as a Cumming estimation plot.

Additional file 6: **Figure S5.** Gene expression analysis of lipoprotein receptors in ESC-derived RPE cells. RT-qPCR analysis of gene expression in stem cells (day 0), early retinal progenitors (day 7), immature RPE cells with low pigmentation (day 50) and mature RPE cells with high pigmentation (~ day 70) cultured on transwell inserts. Fold change in gene expression at different stages of in vitro differentiation as compared to expression in the day 0 cells are shown as Cumming estimation plots. Each plot depicts the data for the indicated gene. The raw data is plotted on the upper axes. On the lower axes, mean differences are plotted as bootstrap sampling distributions. Each mean difference is depicted as a dot. Each 95% confidence interval is indicated by the ends of the vertical error bars.

Additional file 7: **Figure S6.** Gene expression data from the full list of lipoprotein receptors tested in ESC-derived RPE cells. RT-qPCR analysis of gene expression in immature RPE cells with low pigmentation (day 50) and mature RPE cells with high pigmentation (~ day 70) cultured on transwell inserts. Data are presented as target gene expression relative to the mean of three housekeeping genes expression.

Additional file 8: **Figure S7.** TEER values of AcLDL negative and positive population plated after cell sorting. TEER values were measured at day 1, 20 and 45 using an EVOM2 voltohmmeter. The mean difference in TEER values of DiI AcLDL positive (+) and negative (−) cells over time (D0, 20 and 45) in culture is shown as a Cumming estimation plot. The raw data is plotted on the upper axes; each mean difference is plotted on the lower axes as a bootstrap sampling distribution. Mean differences are depicted as dots; 95% confidence intervals are indicated by the ends of the vertical error bars.

## Abbreviations

AcLDL: Acetylated low-density lipoprotein
AMD: Age-related macular degeneration
BSA: Bovine serum albumin
Dil: Di-alkyl indocarbocyanine
DMEM: Dulbecco’s modified Eagle’s medium
DPBS: Dulbecco’s phosphate-buffered saline
ELISA: Enzyme-linked immunosorbent assay
FACS: Fluorescence-activated cell sorting
FITC: Fluorescein isothiocyanate
GAPDH: Glyceraldehyde 3-phosphate dehydrogenase
GMP: Good manufacturing practice
GPI: Glycosylphosphatidylinositol
hESC: Human embryonic stem cells
hiPSC: Human-induced pluripotent stem cells
HMBS: Hydroxymethylbilane synthase
hPSC: Human pluripotent stem cells
KO serum replacement: KnockOut serum replacement
LDLR: Low-density lipoprotein receptor
LHX2: LIM/homeobox protein Lhx2
LRP1: Low-density lipoprotein receptor-related protein 1
PAX6: Paired box 6
PBS: Phosphate-buffered saline
POS: Photoreceptor outer segment
RAX: Retinal homeobox protein Rx
RPE PLUS: RPE purification by lipoprotein uptake-based sorting
RPE: Retinal pigment epithelium
RT-qPCR: Reverse transcription-quantitative polymerase chain reaction
SCARA3: Scavenger Receptor Class A Member 3
SCARB2: Scavenger Receptor Class B Member 2
SCARD1: Scavenger Receptor Class D Member 1
SEM: Standard error of the mean
SIX3: SIX homeobox 3
TEER: Transepithelial electrical resistance
VEGF: Vascular endothelial growth factor
VLDLR: Very low-density lipoprotein receptor
ZO-1: Zonula occludens-1
