# Supplementary material for: Rapid generation of purified human RPE from pluripotent stem cells using 2D cultures and lipoprotein uptake-based sorting
Source: Stem Cell Res Ther. 2020 Feb 3;11:47. doi: 10.1186/s13287-020-1568-3 (PMC6998340; doi:10.1186/s13287-020-1568-3)
Supplement: Supplementary file 1 — Table S1. List of qPCR primers. [file 13287_2020_1568_MOESM1_ESM.pdf]

Table S1

	Forward Sequence	Reverse Sequence
**CD136**	GTCAATGGGACTGAGTGTCTGC	TCTCTGTACTGGAAGGTCCAGG
**CD14**	CTGGAACAGGTGCCTAAAGGAC	GTCCAGTGTCAGGTTATCCACC
**CD207**	TAATCTGCCTGACGCTGGTCCT	GGTGCTGATGTTGTCCACACGA
**CD209**	GCAGTCTTCCAGAAGTAACCGC	GCTCTCCTCTGTTCCAATACTGC
**CD36**	CAGGTCAACCTATTGGTCAAGCC	GCCTTCTCATCACCAATGGTCC
**CXCL16**	CCTATGTGCTGTGCAAGAGGAG	CTGGGCAACATAGAGTCCGTCT
**GAPDH**	GTCTCCTCTGACTTCAACAGCG	ACCACCCTGTTGCTGTAGCCAA
**GPI**	CTGGTAGACGGCAAGGATGTGA	TCCGTGATGGTCTTGCCTGTGT
**HMBS**	ACGGCTCAGATAGCATACAAGAG	GTTACGAGCAGTGATGCCTACC
**LDLR**	GAATCTACTGGTCTGACCTGTCC	GGTCCAGTAGATGTTGCTGTGG
**LHX2**	ACGCCAAGGACTTGAAGCAGCT	TTTCCTGCCGTAAGAGGTTGCG
**LRP1**	CAACGGCATCTCAGTGGACTAC	TGTTGCTGGACAGAACCACCTC
**LRP1B**	ATCGACTACCGTGCAGAGAAGC	CAGCCAAACTGAGGAAAGTCCC
**LRP2**	GTCTAACCGCACTGTGATAGCC	CGGAAGTTTCCTCCCAATGTGG
**LRP4**	GTGTGGCAGAACCTTGACAGTC	ACCGCTCTAACTTGGCATTCTCC
**LRP8**	GAAGCGGAACTATTCACGCCTC	ATGAGGACCTCCTGCTCTTTCG
**LY75**	GCAAGCGATACCTGTCACTCCT	ATAGGCAGTCCAGCGCAAACCA
**MARCO**	GGACAATTTGCGATGACGAGTGG	CCGACACTGAACATTATCCAGCC
**MEGF10**	TGACTGCTTGCCTGGCTTCACA	GTTACAGGTTCCGTTGTTGGTGC
**MRC1**	AGCCAACACCAGCTCCTCAAGA	CAAAACGCTCGCGCATTGTCCA
**MSR1**	TGCACAAGGCAGCTCACTTTGG	GTGCAAGTGACTCCAGCATCTTC
**OLR1**	GAAACCCTTGCTCGGAAGCTGA	CAGATCCAGTCTTGCGGACAAG
**PAX6**	CTGAGGAATCAGAGAAGACAGGC	ATGGAGCCAGATGTGAAGGAGG
**RAGE**	CACCTTCTCCTGTAGCTTCAGC	AGGAGCTACTGCTCCACCTTCT
**RAX**	GCAAGGTCAACCTACCAGAGGT	GCAGCTTCATGGAGGACACTTC
**SCARA3**	CTCCGAAGACATCTCCTTGACC	CCAGCTTCATGGCAGAAAGAGC
**SCARA4**	AGACTCCAAGCATGGTCAGCTC	CCTTTCTGTCCCTTGTTGCCAG
**SCARA5**	GCTGAACCTGTGTGAGGATGGT	TCAGGAAGACCAGCAGGTAGAG
**SCARB1**	GGTCCAGAACATCAGCAGGATC	GCCACATTTGCCCAGAAGTTCC
**SCARB2**	GCCAATACGTCAGACAATGCCG	CTCATCTGCTTGGTAAAAGTGTGG
**SCARD1**	CGAGCATCATTCTTTCACCAGCT	ATGAGAGGCAGCAAGATGGACC
**SCARF1**	AGCTACCGTGTCCAGGATGAAG	GGCTCGATGAAGCTGTGGTTGA
**SCARF2**	CTTTCGCTTGGGAGGAAGAAGG	GTGTGTTATCCAGGTCGTGGTG
**SIX3**	CTCCCACACAAGTAGGCAACTG	TCTGCCGAGCCGTGCGTGGG
**SSC4D**	TCACTACGAGGATGTGGCTGTC	TTCCATTCGGCAGTGTCGTAGG
**SSC5D**	TGTGACTGCCAGTGTTCTGGAG	GGAGTGTTTCGTGGTTGGCATC
**STAB1**	GAACCATGTGCCACTGGAAGGC	AGCGGAATCTCCTGGTGCAGTT
**STAB2**	ACTGGCTCCTTACCAAACCTGC	GAGCAAACACTGTGTAGGCATCG
**VLDLR**	CAAGGATGGCAGTGATGAGGTC	CTCGGATACCATTACACTGCCTG
