# Supplementary figures and images for: Rapid generation of purified human RPE from pluripotent stem cells using 2D cultures and lipoprotein uptake-based sorting
Source: Stem Cell Res Ther. 2020 Feb 3;11:47. doi: 10.1186/s13287-020-1568-3 (PMC6998340; doi:10.1186/s13287-020-1568-3)

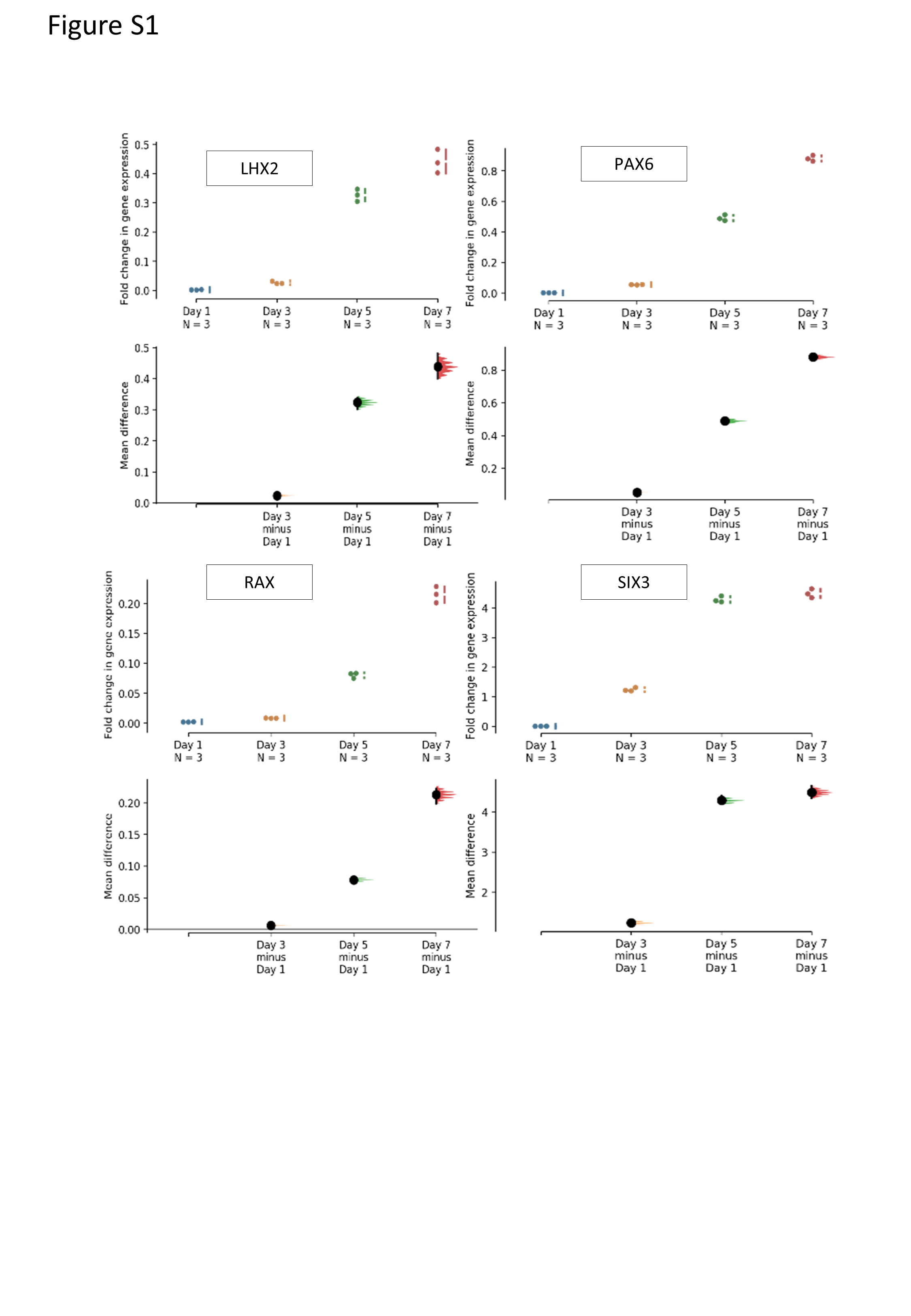

Supplement: Supplementary file 2 — Figure S1. RT-qPCR gene expression analysis of genes involved in early retinal identity. The mean difference in expression of each gene at day 3, 5 and 7 of differentiation is compared against the shared control Day 1 and shown as Cumming estimation plots. The raw data is plotted on the upper axes. On the lower axes, mean differences are plotted as bootstrap sampling distributions. Each mean difference is depicted as a dot. Each 95% confidence interval is indicated by the ends of the vertical error bars. [file 13287_2020_1568_MOESM2_ESM.tif]

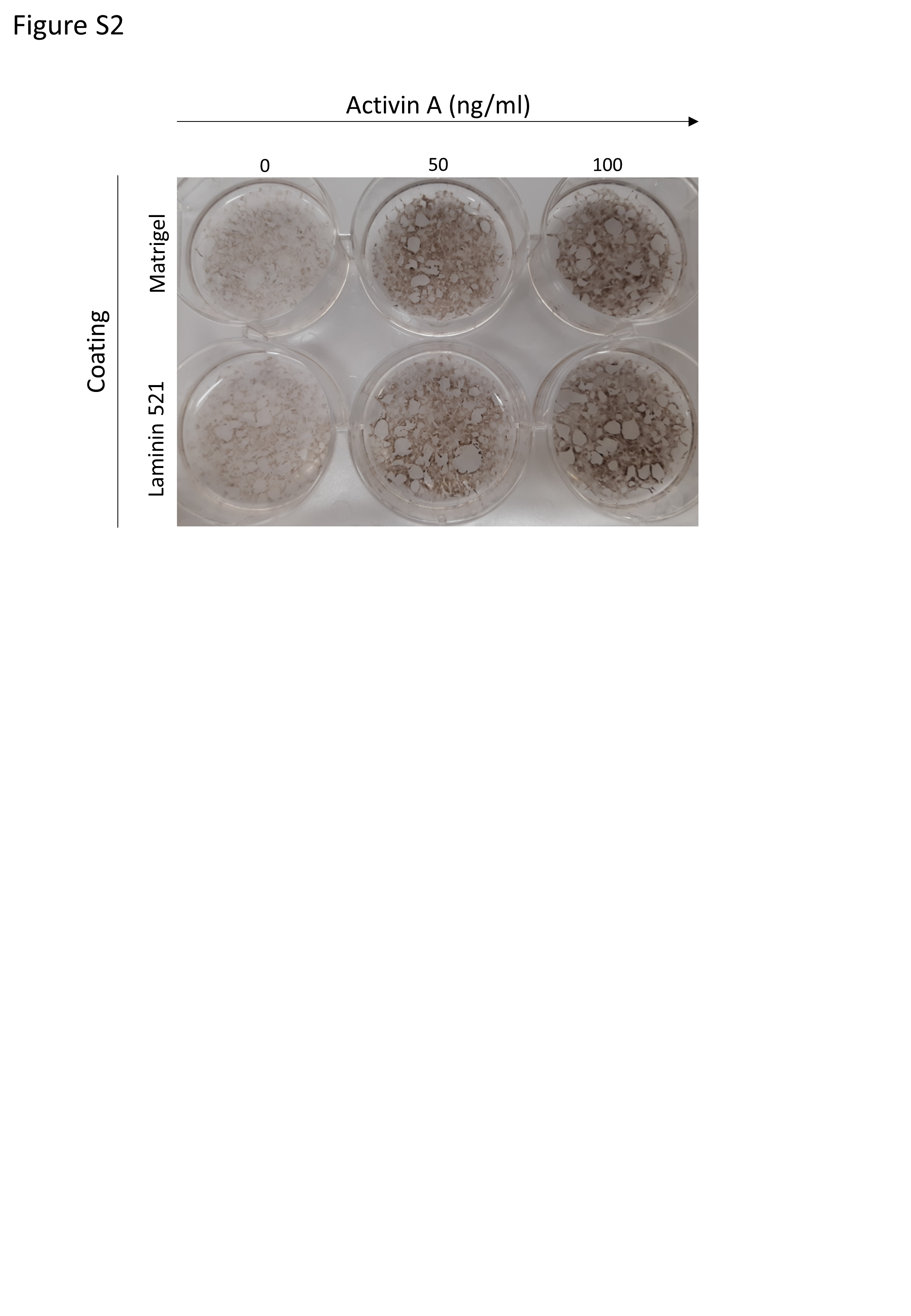

Supplement: Supplementary file 3 — Figure S2. Differentiation of hIPSC into RPE cells under different experimental conditions. Representative image of Day 35 hiPSC line GM23280A differentiated to RPE in a 6-well plate under different experimental conditions. GM23280A cells were cultured on Matrigel-coated plates in mTesrI medium. The last passage prior to differentiation experiment was done in a plate coated with Matrigel or Laminin 521 following manufacturer recommendations. Cells were cultured in mTesrI until confluence and then switched to neuronal induction medium N2B27 for 7 days. After the 7 days neural induction cells were cultured in RPE medium supplemented with 0, 50, 100 ng/ml human Activin A. Pigmentation is indicative of RPE differentiation and maturation. [file 13287_2020_1568_MOESM3_ESM.tif]

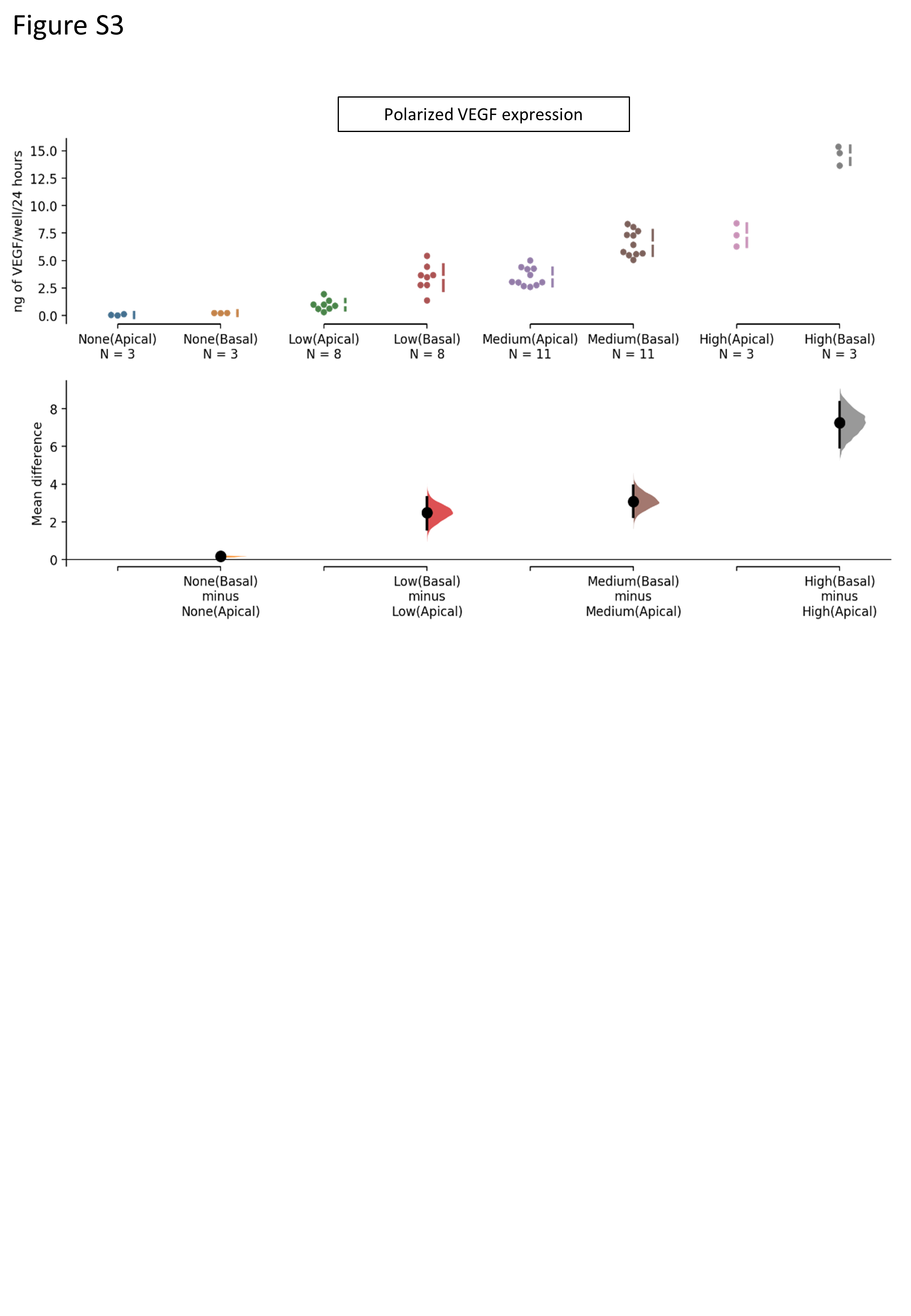

Supplement: Supplementary file 4 — Figure S3. Polarized VEGF secretion assay. ELISA VEGF secretion by hESCs-RPE (H1) on the apical and basal side of a 6.5 mm transwell insert over a period of 24 h. The apicobasal VEGF secretion for each of the 4 samples with varying levels of cell pigmentation are shown in the Cumming estimation plot. [file 13287_2020_1568_MOESM4_ESM.tif]

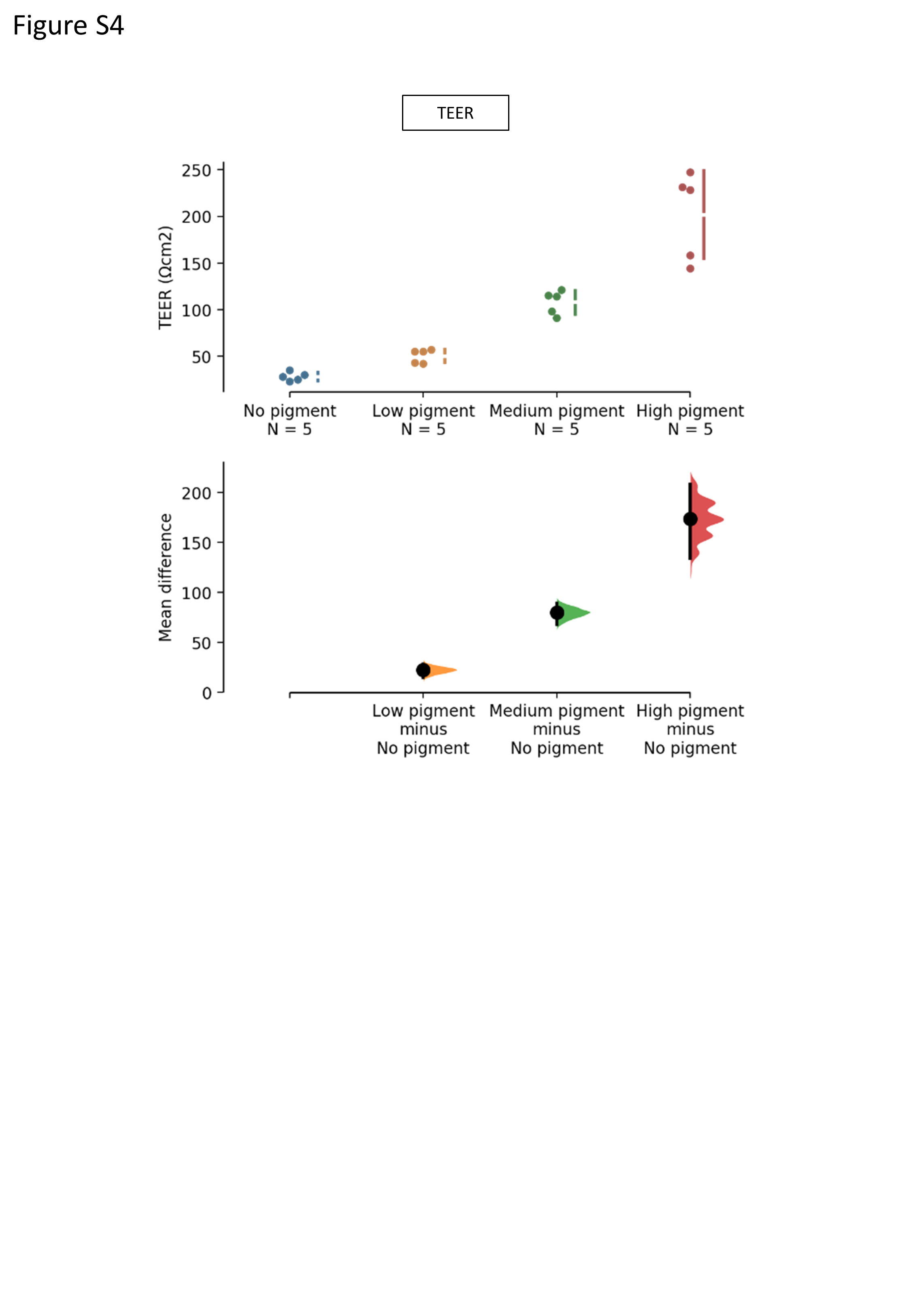

Supplement: Supplementary file 5 — Figure S4. Trans Epithelial Electrical Resistance (TEER) assay. Assessment of TEER of hESCs sheets on transwell inserts during RPE differentiation. The comparison of TEER in increasingly pigmented cells against cells with no pigmentation are shown as a Cumming estimation plot. [file 13287_2020_1568_MOESM5_ESM.tif]

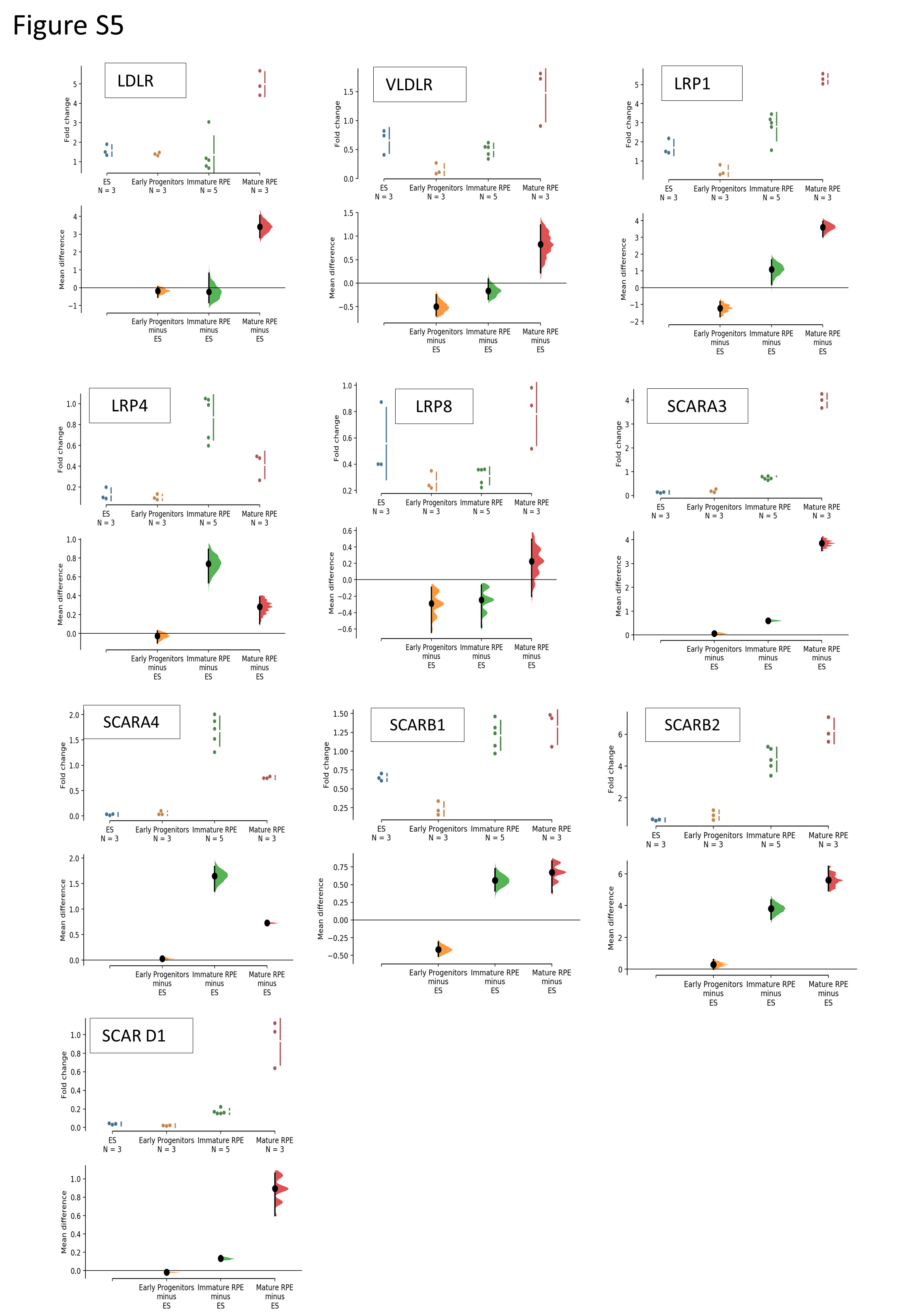

Supplement: Supplementary file 6 — Figure S5. Gene expression analysis of lipoprotein receptors in ESC-derived RPE cells. RT-qPCR analysis of gene expression in stem cells (day 0), early retinal progenitors (day 7), immature RPE cells with low pigmentation (day 50) and mature RPE cells with high pigmentation (~ day 70) cultured on transwell inserts. Fold change in gene expression at different stages of in vitro differentiation as compared to expression in the day 0 cells are shown as Cumming estimation plots. Each plot depicts the data for the indicated gene. The raw data is plotted on the upper axes. On the lower axes, mean differences are plotted as bootstrap sampling distributions. Each mean difference is depicted as a dot. Each 95% confidence interval is indicated by the ends of the vertical error bars. [file 13287_2020_1568_MOESM6_ESM.tif]

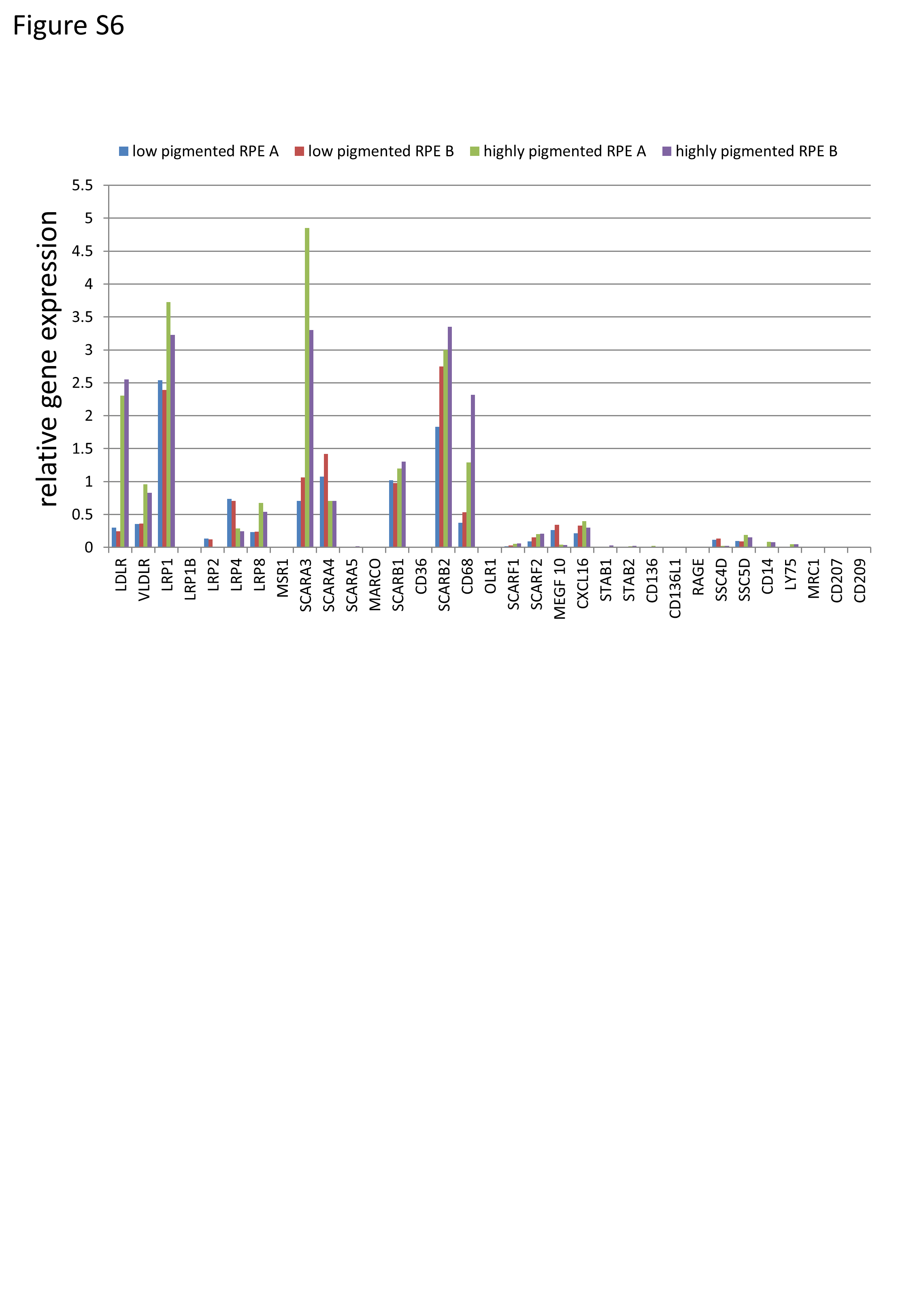

Supplement: Supplementary file 7 — Figure S6. Gene expression data from the full list of lipoprotein receptors tested in ESC-derived RPE cells. RT-qPCR analysis of gene expression in immature RPE cells with low pigmentation (day 50) and mature RPE cells with high pigmentation (~ day 70) cultured on transwell inserts. Data are presented as target gene expression relative to the mean of three housekeeping genes expression. [file 13287_2020_1568_MOESM7_ESM.tif]

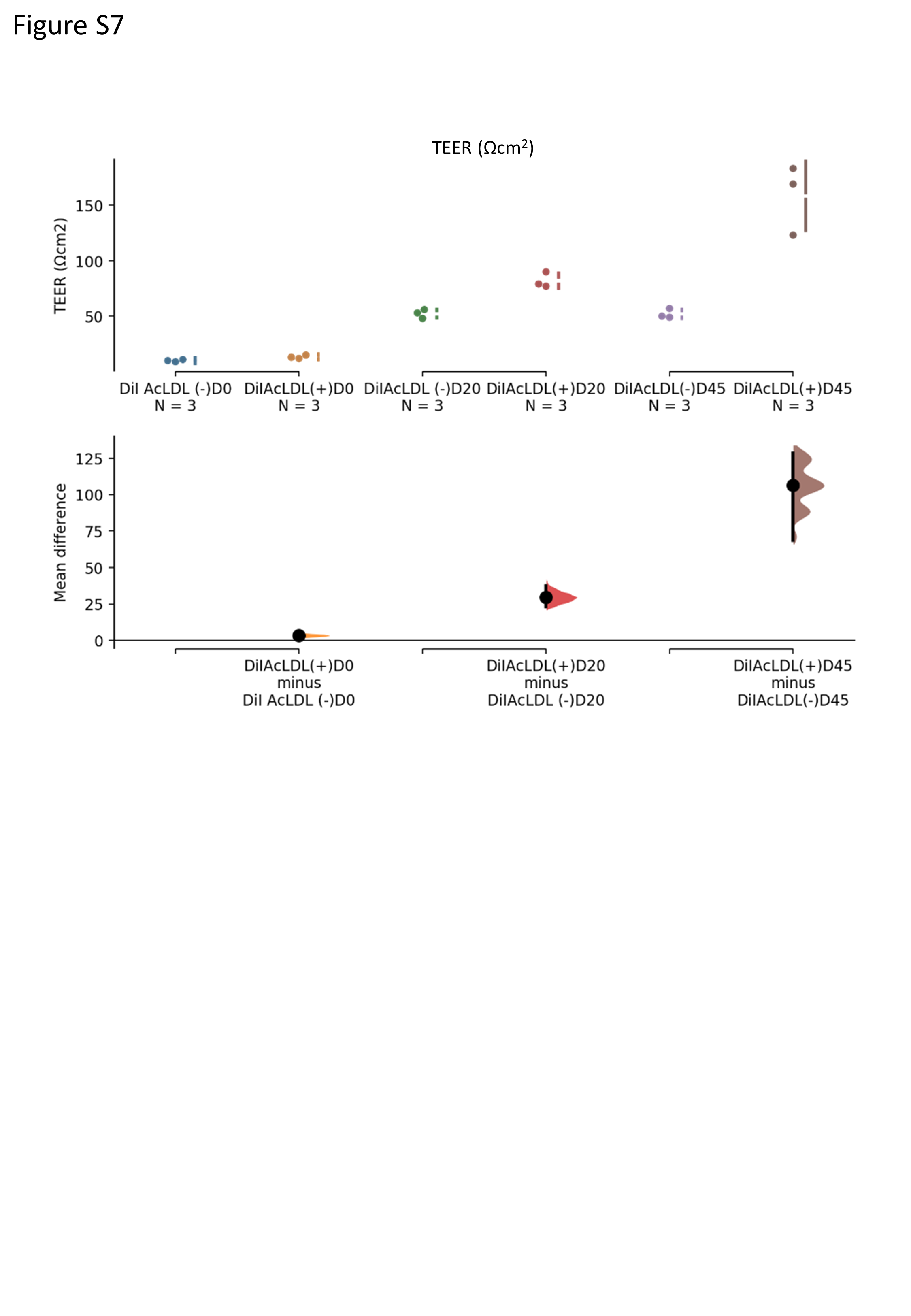

Supplement: Supplementary file 8 — Figure S7. TEER values of AcLDL negative and positive population plated after cell sorting. TEER values were measured at day 1, 20 and 45 using an EVOM2 voltohmmeter. The mean difference in TEER values of DiI AcLDL positive (+) and negative (−) cells over time (D0, 20 and 45) in culture is shown as a Cumming estimation plot. The raw data is plotted on the upper axes; each mean difference is plotted on the lower axes as a bootstrap sampling distribution. Mean differences are depicted as dots; 95% confidence intervals are indicated by the ends of the vertical error bars. [file 13287_2020_1568_MOESM8_ESM.tif]
